# Electrolyte imbalance causes suppression of NK and T cell effector function in malignant ascites

**DOI:** 10.1186/s13046-023-02798-8

**Published:** 2023-09-08

**Authors:** Antonio Hrvat, Mathias Schmidt, Bernd Wagner, Denise Zwanziger, Rainer Kimmig, Lothar Volbracht, Sven Brandau, Nina Mallmann-Gottschalk

**Affiliations:** 1 Experimental and Translational Research, Department of Otorhinolaryngology, University Hospital Essen, Hufelandstrasse 55, 45147 Essen, Germany; 2 Department of Clinical Chemistry, University Hospital Essen, 45147 Essen, Germany; 3 Department of Endocrinology, Diabetes and Metabolism, University Hospital Essen, 45147 Essen, Germany; 4 Department of Gynecology and Obstetrics, University Hospital Essen, 45147 Essen, Germany; 5 partner site Essen-Düsseldorf, German Cancer Consortium (DKTK), 45147 Essen, Germany; 6 Department of Gynecology and Obstetrics, University Hospital of Cologne, 50931 Cologne, Germany

**Keywords:** NK cell, Ascites, Immunosuppression, Sodium, Ovarian cancer, T cell, Channel blocker, Amiloride, Chloride, Electrolyte

## Abstract

**Background:**

Malignant ascites commonly occurs in advanced or recurrent stages of epithelial ovarian cancer during peritoneal carcinomatosis and is correlated with poor prognosis. Due to its complex composition of cellular and acellular components malignant ascites creates a unique tumor microenvironment, which mediates immunosuppression and promotes progression of disease. However, the immunosuppressive mechanisms remain poorly understood.

**Methods:**

In the present study, we explored the antitumor activity of healthy donor NK and T cells directed against ovarian cancer cells in presence of malignant ascites derived from patients with advanced or recurrent peritoneal carcinomatosis. A wide range of methods was used to study the effect of ascites on NK and T cells (FACS, ELISA, EliSpot, qPCR, Live-cell and confocal microscopy, Western blot and electrolyte flux assays). The ascites components were assessed using quantitative analysis (nephelometry, potentiometry and clinical chemistry) and separation methods (dialysis, ultracentrifugal filtration and lipid depletion).

**Results:**

Ascites rapidly inhibited NK cell degranulation, tumor lysis, cytokine secretion and calcium signaling. Similarly, target independent NK and T cell activation was impaired in ascites environment. We identified imbalanced electrolytes in ascites as crucial factors causing extensive immunosuppression of NK and T cells. Specifically, high sodium, low chloride and low potassium content significantly suppressed NK-mediated cytotoxicity. Electrolyte imbalance led to changes in transcription and protein expression of electrolyte channels and impaired NK and T cell activation. Selected inhibitors of sodium electrolyte channels restored intracellular calcium flux, conjugation, degranulation and transcript expression of signaling molecules. The levels of ascites-mediated immunosuppression and sodium/chloride/potassium imbalance correlated with poor patient outcome and selected molecular alterations were confirmed in immune cells from ovarian cancer patients.

**Conclusion:**

Our data suggest a novel electrolyte-based mechanism of immunosuppression in malignant ascites of patients with peritoneal carcinomatosis. We show for the first time that the immunosuppression of NK cytotoxicity in coculture assays is correlated to patient poor survival. Therapeutic application of sodium channel inhibitors may provide new means for restoring immune cell activity in ascites or similar electrolyte imbalanced environments.

**Supplementary Information:**

The online version contains supplementary material available at 10.1186/s13046-023-02798-8.

## Background

Advanced epithelial ovarian cancer is still the gynecological malignancy with worst prognosis [1]. Due to limited efficacy of standard chemotherapies in advanced stages, novel approaches are urgently needed. Because ovarian cancer is considered to be an “immunogenic tumor” promising immunotherapeutic approaches have been conceptualized [2, 3]. Since advanced ovarian cancer usually spreads locally with formation of peritoneal carcinomatosis, intraperitoneal application of cellular and antibody-based immunotherapies is of specific interest [4]. However, until now, such approaches often showed only moderate clinical benefit [5]. Off all epithelial cancers that cause peritoneal carcinomatosis, advanced ovarian cancer is most commonly associated with the formation of malignant ascites, which limits patients´ quality of life and correlates with poor survival [6, 7]. Ascites composition is heterogenous consisting of cancer cells, associated fibroblasts, immune cells and various acellular components that create a proinflammatory environment [8]. Natural killer (NK) cells, innate immune cells and key effectors in antitumor defense, can be isolated in a high proportion in malignant ascites [9]. NK induce natural or antibody-dependent cytotoxicity (ADCC) and secrete antitumoral cytokines like IFNγ or TNFα. However, their functionality in malignant ascites seems to be substantially impaired [10]. Particularly, their cytolytic activity is decreased due to downregulation of Fcγ-receptor CD16 and activating receptor DNAM-1 [11]. Similarly, NK cell-tumor cell-interaction is disturbed since expression of NKG2D is downregulated by soluble MICA and MICB released from adjacent tumor cells [12]. It has been hypothesized that acellular components of malignant ascites contribute to ascites-mediated immunosuppression. Cytokines, proteins, lipids, metabolites and nucleic acids dynamically interact with cellular components of ascites leading to progressive disease [13]. While many studies focused on factors like VEGF and IL6 promoting tumor growth and metastasis [14, 15], only limited data is available about factors which potentially suppress the function of NK cells and other immune effector cells. Among these, IL18 and TGFβ could be identified to suppress NK-mediated ADCC by downregulating CD16 [16]. TGFβ also decreased expression of NK activating receptors NKp30 and NKG2D, whose expression was additionally inhibited by macrophage migration inhibitory factor (MIF) [17, 18]. Among non-cytokine factors MUC16 (CA125) was shown to impair NK cell-mediated ADCC and conjugation [19]. In other studies low molecular proteins were considered to be inhibitory [20].

This study aimed to identify novel acellular factors and mechanisms of NK suppression in malignant ascites. To this end, we studied structural and functional changes of NK cells directed against ovarian cancer cells in presence of malignant ascites after depletion of cellular components. In order to uncover potential candidates, we correlated the content of different electrolytes and proteins in the acellular ascitic fluid to cytotoxic and secretory NK functions and to the functionality of activated T cells. With this approach, we identified a sodium-chloride-potassium imbalance in ascites as a major immune suppressive mechanism that impairs effector functions of NK cells and T cells.

## Methods

### Patient ascites and healthy donor serum sample preparation

Ascites samples from 28 adult patients were collected at the Departments of gynecology and obstetrics at the University Hospital Cologne, Cologne and the University Hospital Essen, Essen, Germany. The statistical analysis of reported survival showed a median survival of 12.13 months (range from 5 days to 74 months) (Supplementary table S1, Additional File 1.). The conduction of the study has been approved by the local Ethics Committee of the medical faculties of Duisburg-Essen and Cologne. The ascites samples were collected during clinically indicated cytoreductive surgery or palliative surgery after written information and consent. The isolated ascites was transferred to Falcon 50 tubes and centrifuged at 2000 g for 10 min. After centrifugation only acellular supernatant was collected. T cells from two patient ascites samples were isolated. The collected fraction was sterile filtered using bottle top filters (Sarstedt, 0.2 μm), aliquoted and frozen at -80 °C for long-term storage. Healthy donor serum was obtained using clotting activator serum collection tubes (Sarstedt). Samples were centrifuged 2000 g for 10 min.

### Human cancer cell lines and in vitro cell culture

For in vitro killing and activation assays, several EGFR-positive cell line models were used:

The human ovarian cancer cell line IGROV1 was kindly provided by LIMES Institute, University of Bonn, Bonn, Germany. The lung cancer cell line A549 (adenocarcinoma) cell line was obtained from Westdeutsches Tumorzentrum, University Hospital Essen, Essen, Germany. The colorectal cancer cell line A431 was obtained from DSMZ, Braunschweig, Germany and FaDu head and neck cancer cell line from LGC Standards, Wesel Germany. All used cancer cell lines showed positive expression for EGFR (epidermal-growth-factor-receptor). IGROV1, A549, A431 and FaDu cell lines were cultured in Roswell Park Memorial Institute (RPMI Gibco) supplemented with 10% (v/v) heat-inactivated fetal calf serum (FCS Gibco), 100 U/mL penicillin, and 100 mg/mL streptomycin (PenStrep, Gibco by Life Technologies) (supplemented complete media). Cells were cultivated in plastic flask (Sarstedt) at 37 °C and 5% CO_2_ and continuously passaged by treatment with Accutase (Gibco) for 5 min at 37 °C. Cells are regularly tested for mycoplasma and STR Typing was performed by the Leibniz-Institute DSMZ Molecular Biology Group.

### NK and T cell isolation from peripheral blood

Healthy donor blood collected in trisodium citrate blood collection tubes (Sarstedt) diluted with Dulbecco’s Phosphate Buffered Saline (DPBS, Gibco, Life Technologies Limited) in a 1:1 ratio and overlayed on a 1.077 g/mL separation medium (Biocoll, Merck Millipore). Density centrifugation was performed at room temperature (400 g for 30 min) without acceleration and brake. Same methodology was used during the isolation of T cells from patient ascites samples. PBMCs were collected and plastic adherence was performed to deplete monocytes by incubating them in a T175 flask (Sarstedt) at 37 °C and 5% CO_2_ for 1 h. For NK isolation, NK MACS Isolation Kit (Miltenyi Biotec) was used according to the manufacturer’s instructions. After isolation 5 U/ml recombinant human IL-15 (50 µg, Immuno Tools) was added to NK cells that were used for functional experiments after incubation overnight. For T cell isolation, CD3 human microbeads (Miltenyi Biotec) were used according to the manufacturer’s instructions. Purity of isolated T cells was routinely tested. In case of overnight resting 5–10 U/ml of recombinant human IL-2 (50 µg, Immuno Tools) was added to T cells.

### NK degranulation assay

NK cells express CD107a during degranulation, which also correlates to NK cell-mediated tumor cell lysis. To evaluate natural and antibody-dependent NK cell cytotoxicity purified NK cells and target cells were coincubated (1:1 ratio) in a flat-bottom 96-well plate with or without different ascites samples. For ADCC-experiments the human anti-EGFR-antibody Cetuximab 1 µg/ml (Erbitux, 5 mg/mL, Merck (Serono)) was added. NK cells were labeled with anti-CD107a-FITC (25 µg/mL, clone H4A3, Mouse IgG1, k, BD Biosciences) and isotype control, respectively. After incubation for 1 h at 37 °C and 5% CO_2_, Golgistop Monensin (BD Biosciences) was added (1:600). After a further incubation of 5 h NK cells were stained with CD56-BV421 (12 µg/mL, NCAM 16.2, IgG2b,k, BD Biosciences) and isotype control. CD107 expression was analyzed by flow cytometry. In case of sodium channel blocker experiment, NK cells were pretreated for one hour with 15 µM of Amiloride after Cell Tracker Red staining.

### NK cell tumor killing assay

NK cells and target cells were coincubated (1:1 ratio) with or without Cetuximab (1 µg/ml) and different ascites samples in resulting ratio 1:4 for 24 h at 37 °C and 5% CO_2_. Adherent and suspended cells were harvested with Stem Pro Accutase (Gibco by Life Technologies). After washing cells were stained using PE Annexin V Apoptosis Detection Kit I (BD Biosciences) according to the manufacturer’s protocol and analyzed by flow cytometry. Alive cells were defined as Annexin V-/7AAD.

### Flow cytometric analysis of NK, T cell and tumor cell markers

After coincubation with 25% supplemented ascites media for 24 h, the following antibodies for the flow cytometric analysis of the NK cell marker expression were used: CD56-BV421 (12 µg/mL, clone NCAM 16.2, mIgG2b,k, BD Biosciences), NKp46-PE (CD335, 50 µg/ml, clone 9E2, mIgG1, k, Biolegend), DNAM-1-PerCP-Cy5.5 (CD226, 200 µg/ml, clone 11A8, mIgG1, k, Biolegend), TIGIT-APC (30 µg/mL, A15153A, mouse IgG2b,k, BioLegend), NKG2D-PE-Cy7 (CD314, 200 µg/ml, clone 1D11, mIgG1, k, Biolegend), CD69-FITC (100 µg/ml, clone FN50, mIgG1, k, Biolegend), followed by a live/dead staining using the fixable viability dye eFluor 780 (eBioscience/Thermo Fisher Scientific, Darmstadt, Germany). After coincubation with 25% supplemented ascites media for 48 h, the following antibodies for the flow cytometric analysis of the T cell marker expression were used: CD4-AF647 (100 µg/mL, OKT4, mouse IgG2b,k, BioLegend), CD25-PE (44 µg/mL, 4 E3, mouse IgG2b,k, Miltenyi), CD8-VioBlue (50 µg/mL, LT8, mouse IgG1,k, Miltenyi), Tim-3-PerCP-eFlour710 (12 µg/mL. F38-2E2, mouse IgG1,k, eBioscience). Ovarian cancer cell surface marker expression were detected by staining with the following antibodies: MICA-APC (5 µg/ml, clone 159,227, mIgG2b, k, RD Systems), UBLP-2/5/6-APC (10 µg/ml, clone 165,903, mIgG2a, k, RD Systems), CD54-PE (100 µg/ml, clone HA58, mIgG1,k, Biolegend), and MHCI-PE (25 µg/ml, clone W6/32, mIgG2a,k, Biolegend), HLA-E-APC (200 µg/mL, 3D12, mouse IgG1,k, Biolegend), CD112-PerCP-Cy5.5 (Nectin-2, 200 ug/ml, TX31, mouse IgG1,k, BioLegend), CD155-FITC (PVR1, 100 ug/ml, SKII.4, mouse IgG1,k, BioLegend), CD178-PE (Fas-L, 200 µg/mL, NOK-1, mouse IgG1,k, Biolegend), CD48-VioBright FITC (REA(S)293, mouse IgG1,k, Miltenyi), EGFR-PE (6.3 ug/ml, EGFR.1, mouse IgG2b,k, BD Bioscience). The same fixable viability dye as for NK markers was used. In all flow cytometry measurements appropriate isotype controls were used: mIgG1-BV510 (100 µg/ml, clone MOPC-21, Biolegend), mIgG1-PE (50 µg/ml, clone MOPC-21, BD Bioscience), mIgG1-PE-Cy-7 (200 µg/ml, MOPC-21, Biolegend), mIgG1-FITC (500 µg/ml, clone MOPC-21, Biolegend), mIgG1-PerCP-Cy5.5 (200 µg/ml, clone MOPC-21, Biolegend), mIgG1-APC (200 µg/ml, clone MOPC-21, Biolegend), mIgG2b-APC (200 µg/ml, clone MPC-11, Biolegend), mIgG2a-APC (200 µg/ml, clone MOPC-173, Biolegend), mIgG1-PE-Cy7 (200 µg/mL, MOPC-21, BioLegend), mIgG1-PerCP-eFlour710 (200 µg/mL, P3.6.2.8.1, eBioscience), mIgG2a-PE (50 µg/mL, G155-178, BD Bioscience), mIgG2a-PerCP-Cy5.5 (200 µg/mL, MOPC-173, BioLegend), mIgG2b-AF647 (200 µg/mL, MOPC-173, BioLegend), mIgG2b-Alexa Fluor®405 (50 µg/mL, 133,303, R&D Systems), mIgG2b-PE (200 µg/mL, 27–35, BD Biosience).

Stained cells were analyzed with BD FACS Canto II using DIVA 8.01 software (BD Biosciences) or FlowJo10 (LLC, Ashland, Oregon, USA).

### Detection of NK IFNγ secretion by ELISpot

The ELISpot-technique was applied for sensitive detection of IFNγ-secreting NK cells. First, a Multiscreen 96-well filtration plate (Merck Millipore) was activated with 35% ethanol and coated with anti-IFNγ capture antibody (200 µg/ml, clone 1-D1K, mIgG1, k, Mabtech). After incubation at 4 °C overnight, the plates were blocked with 200 µl of supplemented complete RPMI-1640-media for 2 h at 37 °C. After the washing step isolated NK cells were coincubated with IGROV1 cells in 1:1 ratio and either with or without ascites samples (ratio 1:4). For ADCC conditions, Cetuximab 1 µg/ml (Erbitux, 5 mg/mL, Merck (Serono) was added. After incubation for 24 h at 37 °C and 5% CO_2_ plates were washed in the ELISA-washer (PBS / 0,05% Tween-20.) Biotinylated anti-IFNγ detection antibody (200 µg/ml, clone 7-B6-1, mIgG1, k, Mabtech) was added in 2 µg/ml PBS and 1% BSA. The plates were incubated for 2 h at 37 °C, washed and incubated with 50 µl ExtraAvidin alkaline phosphatase (1:1000 diluted in PBS / 1% BSA, Sigma-Aldrich) at room temperature for 2 h. After the washing steps, 75 µl of the ELISpot substrate BCIP/NBT (Roche) was added and incubated for 5–10 min. Developed cytokine spots were measured using AID Classic ELISpot Reader and the results were analyzed with AID ELISpot 7.0 software.

### Detection of T cell IFNγ secretion in culture supernatant by ELISA

100.000 T cells were stimulated by 2,5 µl (1:40 dilution) of CD2/CD3/CD28 activator complex (StemCell Technologies) either in media or 25% ascites supplemented media for four days. After incubation period the experiment supernatant was collected and secreted IFNγ quantified using ELISA kits (R&D Systems, Wiesbaden, Germany) according to the manufacturer’s instructions. The plate was measured using spectrophotometer Synergy2 (BioTek).

### Conjugation assay

The conjugation rate of NK and tumor cells was analyzed in presence of ascites. NK cells were stained with Cell Tracker Red (Thermo Fisher Scientific) and tumor cells with Cell Tracker Green (Thermo Fisher Scientific) according to the manufacturer’s instructions. NKs (50.000 cells) and IGROV1 tumor cells (200.000 cells) were coincubated (1:4 ratio) in presence of 1 µg/ml Cetuximab with or without 25%-ascites-supplemented media. After centrifugation (20 g (570 rpm), 1 min) tubes were placed in a water bath at 37 °C for 45 min. Afterward, the samples were retrieved and briefly vortexed. 300 µl of ice-cold 0,5% PFA in PBS was added and samples were analyzed by flow cytometry. Using flow cytometry, events positive for both cell trackers were determined as conjugates. In case of sodium channel blocker experiment, NK cells were pretreated for one hour with different Amiloride concentrations (1.5, 15 and 150 µM) after Cell Tracker Red staining and dose-response was studied.

### Live cell imaging of tumor killing kinetics

100.000 IGROV1 cells per well were seeded in the 96-well plate overnight. Cells were stained with 10 ug/ml Hoechst (Thermo Fisher Scientific) at 37 °C in PBS, HS 3%. After 30 min. cells were washed in PBS and stained with 1:250 dilution of CalceinAM-Orange Red (Thermo Fisher Scientific) at 37 °C in serumless RPMI 1640 for 30 min. The staining solution was replaced by phenol-free RPMI1640 supplemented with 10% (v/v) heat-inactivated fetal calf serum (FCS Gibco), 100 U/mL penicillin, and 100 mg/mL streptomycin (PenStrep, Gibco by Life Technologies) (supplemented complete media). Cells were imaged with ImageXpress Pico Automated Cell Imaging System (Molecular Devices) using 4x objective. Images were captured for three hours, once every 15 min. Hoechst and CalceinAM-Orange Red were visualized at Ex370/Em450 nm for 40 ms (DAPI) and Ex530/Em594 nm for 90 ms (TRITC), respectively. Cells were cultured in the incubating unit at 37.5 °C and 5% of CO_2_.

### Confocal imaging of PI3K membrane recruitment

50.000 healthy donor T-cells were seeded per well in a 96-well plate. Cells were cocultured either complete media or 25% ascites-supplemented media. T cells were stimulated for 5 min with CD2/CD3/CD28 activator complex (StemCell Technologies) diluted at 1:20 at 37 °C. In the case of sodium blocker experiments, 150 µM of Amiloride (Sigma-Aldrich) was added to the coculture. After stimulation cells were fixed and permeabilized using Cytofix/Cytoperm (BD Biosciences). Following permeabilization, cells were stained with PIK3R1/p85 antibody and corresponding secondary. T cells were stained by following antibodies in confocal microscopy experiments: PIK3R1/p85 (500 µg/ml, Rat IgG2a, k, W16101a, Biolegend), rat-IgG2a (KLH/G2a-1-1, Dianova), donkey anti-rat AF555 (H + L, 2 mg/ml, polyclonal, Invitrogen) The staining and imaging were performed in Permawash (BD Biosciences). Cells were imaged with confocal microscope Zeiss Elyra PS. (Zeiss) using 1.4 NA 60x oil immersion objective. Alexa Fluor 555 was visualized at Ex561/Em579 nm for 3.85 s per frame. Stained cells were analyzed with ImageJ.

### T cell proliferation assay

Isolated healthy donor T-cells were stained using Cell Proliferation Dye EFluor 450 (Thermo Fisher Scientific, 1:1000) for 10 min at 37 °C. For the assay, 50.000 T cells were seeded per well in a 96-well plate. The assay was performed either in complete media or 25% ascites-supplemented media. T cells were stimulated for four days with CD2/CD3/CD28 activator complex diluted at 1:40 (StemCell Technologies). Stained cells were analyzed with BD FACS Canto II using DIVA 8.01 software (BD Biosciences) and FlowJo10 (LLC, Ashland, Oregon, USA).

### Electrolyte flux assay

For assessment of intracellular calcium or sodium flux T-cells were stained using Fluo-4 (Thermo Fisher Scientific) or CoroNa Green (Thermo Fisher Scientific), respectively, according to the manufacturer’s specifications. For every experimental condition, 250.000 prestained T-cells from original suspensions were taken and incubated either in phenol-free media or 25% ascites-supplemented media in 96 well plates. This was done to ensure consistent dye loading and allow for comparison between samples. In the case of sodium blocker experiments, 150 µM of Amiloride (Sigma-Aldrich), Lidocaine (Sigma-Aldrich), Cariporide (Sigma-Aldrich) and Digitoxin (Sigma-Aldrich) were added to the media as well. The baseline was recorded for 1 min and then 10% (%v/v) of CD2/CD3/CD28 activator complex was added to induce T-cell activation. Intracellular calcium flux was measured at 37 °C using spectrophotometer Synergy2 (BioTek) for 10–15 min in intervals of every 3 s. Intracellular calcium flux data were normalized according to the average unstimulated baseline of media or ascites. In the case of intracellular sodium flux experiment was measured at 37 °C for 20–25 min in intervals of every 5–7 s depending on the experiment. Absorbance was recorded at 480/25 for both Fluo-4 and CoroNa Green. Data for every experimental condition was normalized to the appropriate blank (ascites fluid only) control and presented as fold change of starting point measurement. In the case of calcium flux, comparison was made between peak values of the flux. For sodium influx, endpoint value was compared between conditions. ROC curve analysis was used to confirm significance between different measured flux curves.

### Gene expression analysis by quantitative RT–PCR

For quantitative RT–PCR analysis total RNA was isolated from NK and T cells using the RNeasy Mini kit (Qiagen) and reverse transcribed with random-hexamer primer and Superscript II RT, according to the manufacturer’s instructions (Thermo Fisher Scientific). Quantitative real-time PCR was conducted with Luna® Universal qPCR Master Mix (New England Biolabs). Primers are listed in Supplementary Table S2, Additional File 1. Representative signal transduction and ion channel genes from different groups were selected for screening. The selection was done according to their functional importance and expression in NK cells as reported in existing RNAseq databases (Gene Expression Omnibus ID GSE153713, https://www.ncbi.nlm.nih.gov/geo/query/acc.cgi?acc=GSM4650127) [21]. The annealing temperature was 62 °C for all primers. Gene expression was normalized according to the chosen housekeeping gene (NK cells – β2-microglobulin, T cells – β-actin) and referent control sample.

### Clinical chemistry analysis of patient ascites

Concentrations of sodium, potassium and chloride were measured potentiometrically using an ion-selective electrode on the Atellica CH Analyzer (Siemens Healthineers). Determinations of magnesium (xylidyl blue method), phosphate (ammonium molybdate method), calcium (o-cresol phthalein complexone method), glucose (hexokinase method), total protein (pyrogallol red method), albumin (bromocresol green method) were performed photometrically on the Atellica CH Analyzer (Siemens Healthineers). Immunoturbidimetric methods were used in the measurements of Lp(a), Apo A1, and Apo B on the Atellica CH Analyzer (Siemens Healthineers). Immunonephelometric concentration determinations of IgG, IgA, IgM, the IgG subclasses, α1-antitrypsin, α2-macroglobulin and transferrin were performed on the BN-II (Siemens Healthineers). Measurements of IgE-activity and concentrations of CA 125, CA 15 − 3, CA 19 − 9, and CA 72 − 4 were performed via chemiluminescent sandwich immunoassays on the Atellica IM (Siemens Healthineers). Ammonia concentrations were determined on Dimension XPand Plus (Siemens Healthineers) by enzymatic method using glutamate dehydrogenase. Measurements of pH values were performed on the pH meter pH700 (Eutech Instruments). Controls of the instruments were performed according to the manufacturer’s instructions. Parameters are accredited according DIN EN ISO 15189:2014.

### Ultracentrifugation filtration, dialysis and depletion of lipids from ascites samples

Ultracentrifugation filters (Amicon, 0.5 ml, MCWO 3 kDa) were filled up with ascites and centrifuged at 14.000–15.000 g. The centrifugation was stopped for 15 min, after which the remaining ascites in the insert tube was resuspended and centrifugation was continued for 15 min. Upon completion colorless permeate was collected and used without any volume changes or adjustments. For experiments using heated samples permeate was inactivated by heating to 90 °C for 30 min. The volume of the retentate fraction was measured with the pipette during the collection. Subsequently, the retentate was restored to the original volume using phenol-free RPMI 1640. This washing and resuspension of retentate was repeated three times before using the fraction for in vitro testing. For dialysis of ascites samples, dialysis tubing was filled up with ascites and put in beaker containing 1 L of cell culture medium RPMI 1640 (Pur-a-lyzer, Sigma-Aldrich, 1 kDa and 25 kDa). Tubing was left to incubate for 24 h, after which dialyzed sample was retrieved. Delipidation of ascites samples was done via activated charcoal (20/40 mesh, Supelco) stripping according to an existing protocol [22].

### Migration assay

To study the effects of ascites on NK cell migration, 100.000 NK cells in 200 µl of media were added on top of the transwell insert (Sarstedt, 3 μm) that was put onto a 24well plate (Sarstedt). To induce of NK cells the migration, NK cell chemoattractant SDF-1a (Prepotech) was added to the bottom of the transwell assay at 100 ng/ml concentration. Different ascites samples were added as well to the bottom of the transwell in a 1:4 ratio. One well was prepared without chemoattractant to serve as a control for spontaneous migration and another was with added cells but without insert to simulate maximum migration. The migration experiment was incubated at 37 °C for 3 h. After the insert was removed migrated cells were transferred into a 1.5 ml Eppendorf tube. Cells were washed in PBS and resuspended in 200µL of PBS with 3% human serum addition and 25µL of 123count™ eBeads (Thermo Fisher Scientific). Migration was measured by flow cytometry. A stopping gate was set on counting beads (gated on via 488 nm excitation source) and 5.000 beads were acquired in each sample. The migration was calculated according to the following formula: Absolute count (cells/µl) = $\frac{Cellcount+eBeadsvolume}{Cellvolume+eBeadscount}*eBeads concentration$. The absolute count of every condition was normalized to the absolute count of maximum migration control.

### Electrophoresis and western blot

For mechanistic experiments, 10^6^ of NK cells were stimulated for five minutes with 50 ng/ml of PMA and 1 µg/ml of Ionomycin, and lysed with Urea buffer containing 25 mM HEPES (pH 7.3), 0.1% SDS, 1% Triton X-100, 10 mM EDTA, 10 mM sodium pyrophosphate, 10 mM NaF, 125 mM NaCl, 1% protease inhibitor cocktail I, 1% protease inhibitor cocktail III, and 10% PhosStop. Cell debris was removed by centrifugation, and the lysates were incubated with SDS sample buffer (pH 6.8) 50 mM Tris, 4% glycerine, 0.8% SDS, 1.6% β-mercaptoethanol, and 0.04% bromphenol blue) before boiling them at 95 °C for 10 min. 5 µl Spectra Multicolor broad range protein ladder (Thermo Fisher) was included as a molecular marker. The protein of total lysates (18 µl of each cell line) was separated by SDS-7.5%-polyacrylamide gel and immunoblotted according to the semi-dry-blot-method onto polyvinylidene difluoride membrane (PVDM, Roche Diagnostics). The membrane was incubated with the primary antibody followed by HRP-conjugated secondary goat-anti-rabbit IgG. For immunodetection the following antibodies were used: phospho-PI3K-p85/p55 (Cell Signaling Technology), phospho-PLCγ1 (Cell Signaling Technology), phospho-MAPK-p38 (Cell Signaling Technology), Granzyme B (Cell Signaling Technology), PIK3R1/p85 (Rat IgG2a, Biolegend) (Cell Signaling Technology), CLIC1 (Cell Signaling Technology), SLC24A2 (Cell Signaling Technology), GAPDH (total protein) (Cell Signaling Technology, clone 14C10), goat anti-rabbit IgG HRP (Cell Signaling Technology, No. 7074) and goat anti-rat IgG HRP (Dianova). Bands were visualized after the application of the Clarity and Clarity Max ECL Western Blotting substrates (Bio-Rad) and chemiluminescent transformation. The chemiluminescent signal was recorded with the Amersham Imager 600 (GE HealthCare, Chicago, Illinois, USA). Images were analyzed using ImageJ. Phosphorylation was determined by densitometry and normalized to GAPDH and respective ascites and blocker treated controls.

### Statistical analysis

Graph data are shown as single values (either single ascites samples or healthy blood donors), means as center values and error bars for the standard deviation (SD). Data points shown in heatmap represent mean values. Statistical tests are described in details in the respective figure legend. Post hoc tests were used after ANOVA when appropriate. All calculations and statistical tests were performed using GraphPad Prism 8 software. t-SNE analysis was performed using online tool (https://cs.stanford.edu/people/karpathy/tsnejs/csvdemo.html) based on published algorithm [23]. Hierarchical clustering and PCA analysis were performed using online tool ClustVis which was previously published [24].

## Results

### Malignant ascites strongly inhibits anti-tumor NK cell effector function

**Fig. 1:**
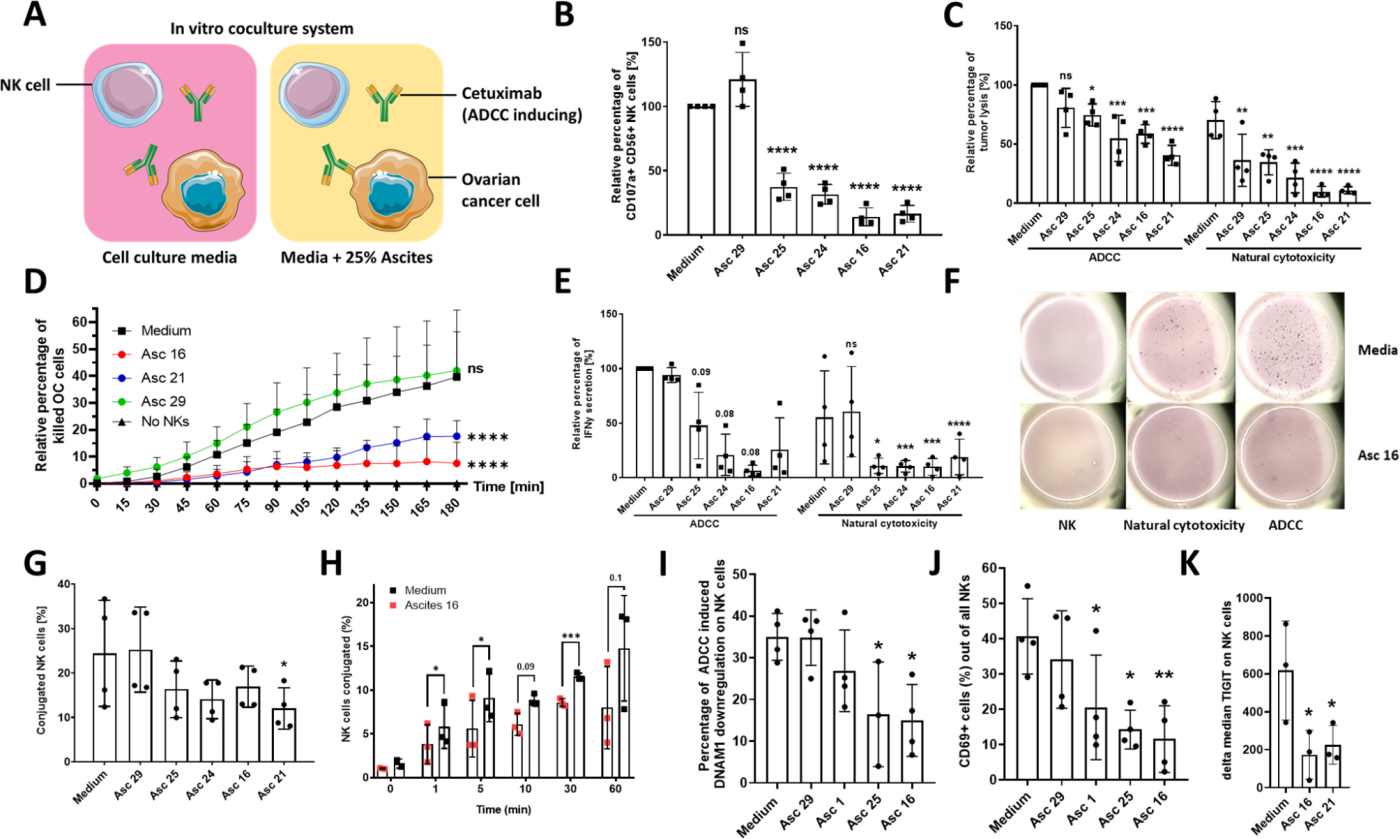
Malignant ascites impairs different NK cell effector functions. **(A)** Graphical illustration portraying components of performed coculture experiments. **(B)** NK-ADCC in presence of ascites. Resting NK cells from healthy donors were coincubated in 1:1 ratio with IGROV1- cells for six hours with addition of ADCC-inducing anti-EGFR-antibody Cetuximab (1 µg/ml) and malignant ascites (No. 16, 21, 24, 25) and benign ascites (No.29), respectively. **(C)** Tumor cell lysis in presence of ascites. Resting NK cells were directed in 1:1 ratio against IGROV1-cells and coincubated with or without Cetuximab and various ascites samples. After 24 h tumor killing was quantified and relative percentage of lysis is shown. **(D)** Time-dependent tumor cell killing. IGROV1 cells cocultured to resting NK cells and Cetuximab were prestained with CalceinAM-OrangeRed and Hoechst. Dead cells were quantified by loss of CalceinAM dye and normalized to control. **(E + F)** IFNγ secretion of NK cells in presence of ascites. **(E)** Relative percentage of IFNγ secreting NK cells in ELISpot assay **(F)** Representative ELISpot-experiment showing IFNγ secretion spots. **(G and H)** Effect of ascites on NK-tumor cell-conjugation. NK and IGROV1-cells were mixed in 4:1 effector to target ratio in presence of Cetuximab and 25%-ascites-supplemented media. **(G)** Percentage of conjugated NK cells measured by flow cytometry after 45 min of coincubation **(H)** Kinetics of conjugation formation. **(I-K)** Expression of NK markers in presence of ascites. NK cells were cocultured (1:1) with IGROV-1-cells and Cetuximab with or without ascites for 24 h. Surface expression of **(I)** DNAM-1, **(J)** CD69, and **(K)** TIGIT was determined using flow cytometry. Each datapoint represents one healthy donor. The relative percentages are shown after normalization to normal medium control. For significance testing ordinary one-way ANOVA (1.B, C, E, G, I-K) and two-way ANOVA (1.D) were performed, followed by Dunnett’s multiple comparison posthoc test between ascites and medium control group where appropriate. Paired t-test was used (1.H). ns (non-significant), * (*p* < 0.05), ** (*p* < 0.01), *** (*p* < 0.001), **** (*p* < 0.0001)

For initial experiments NK cells of healthy donors were coincubated with ovarian cancer cells (IGROV1) with or without 25% ascites supplemented media. Natural cytotoxicity (NC) was assessed by determining the expression of CD107a on NK cells. Antibody-dependent cellular cytotoxicity (ADCC) was induced by addition of the specific antibody Cetuximab directed against EGFR (epidermal growth factor receptor) on target cells (Fig. 1A). Using this setup, we tested the relative inhibitory capacity of ascites samples on NK cell degranulation and killing in both NC and ADCC conditions. Complete characterization of all 28 patient ascites samples regarding their inhibitory potential on NK ADCC degranulation enabled a categorization in very strong, strong, medium, weak inhibitory samples and stimulatory (Table S3, Fig S1A, Additional File 1.). In subsequent functional experiments, we focused on ascites samples which have caused strong or very strong inhibition.

Using this approach, we observed that several malignant ascites samples (no. 25, 24, 21, 16) induced statistically significant inhibition of NK-ADCC after 6 h (Fig. 1B) in a concentration-dependent manner (Fig. S1B). This ascites-mediated immunosuppression of NK cytotoxicity operated over a wide range of ADCC-inducing Cetuximab antibody concentration (Fig. S1C). Benign ascites (no. 29) derived from a patient with congestive heart failure did not restrict NK-ADCC (Fig. 1B). Healthy donor peripheral blood serum was used as an additional control since it is often compared to ascites fluid in regards to its composition and is much easier to obtain then healthy peritoneal fluid. The addition of the serum only marginally reduced NK function (Fig. S1B). The inhibitory effect of malignant ascites also extended to tumor lysis of ovarian cancer cells in presence (ADCC) or absence of Cetuximab (natural cytotoxicity) after 24 h (Fig. 1C), and was already significant after 2 h (Fig. 1D). In order to exclude that suppression of cytotoxic NK cell function is depending on a specific target cell line, we confirmed our results by using other EGFR-positive cell lines (Fig. S1D, E). Further control showed that ascites did neither directly affect NK cell viability (Fig. S1F) nor tumor cell viability (Fig. S2A, Additional File 1). Accordingly, expression of various surface markers on tumor cells, which are considered to be relevant for the interaction between NK and target cells, remained largely unchanged or did not correlate with ascites-induced inhibitory capacity (Fig. S2B-L, Additional File 1.). Our next experiments evaluated the effect of ascites on cytokine secretion by NK cells. Performing ELISpot assay we could observe that secretion of IFNγ of NK cells cocultured to ovarian cancer cells was substantially reduced in presence of malignant ascites while benign ascites did not affect secretory NK cell function. This impairment was demonstrable for NC- and ADCC-condition (Fig. 1E and F). In further experiments, we examined the impact of ascites on the NK-tumor cell-interaction. It could be demonstrated that the conjugation between NK and cocultured ovarian cancer cells in presence of Cetuximab was substantially reduced by malignant ascites but not by benign sample (Fig. 1G), and this effect could be observed early, already after 5 min (Fig. 1H). Similarly, only the presence of malignant ascites inhibited NK cell migration capabilities (Fig. S3A, Additional File 1.). Corresponding, the expression of regulatory and activation NK cell markers like DNAM-1, CD69 and TIGIT were negatively affected only by malignant ascites (Fig. 1I-K).

In conclusion, in these initial experiments we show that malignant ascites derived from patients with ovarian carcinoma or other adenocarcinoma but not benign ascites causes substantial suppression of major antitumoral NK cell effector functions during interaction with various EGFR-positive target cells.

### Malignant ascites interferes with in vitro activation of NK and T cells

**Fig. 2:**
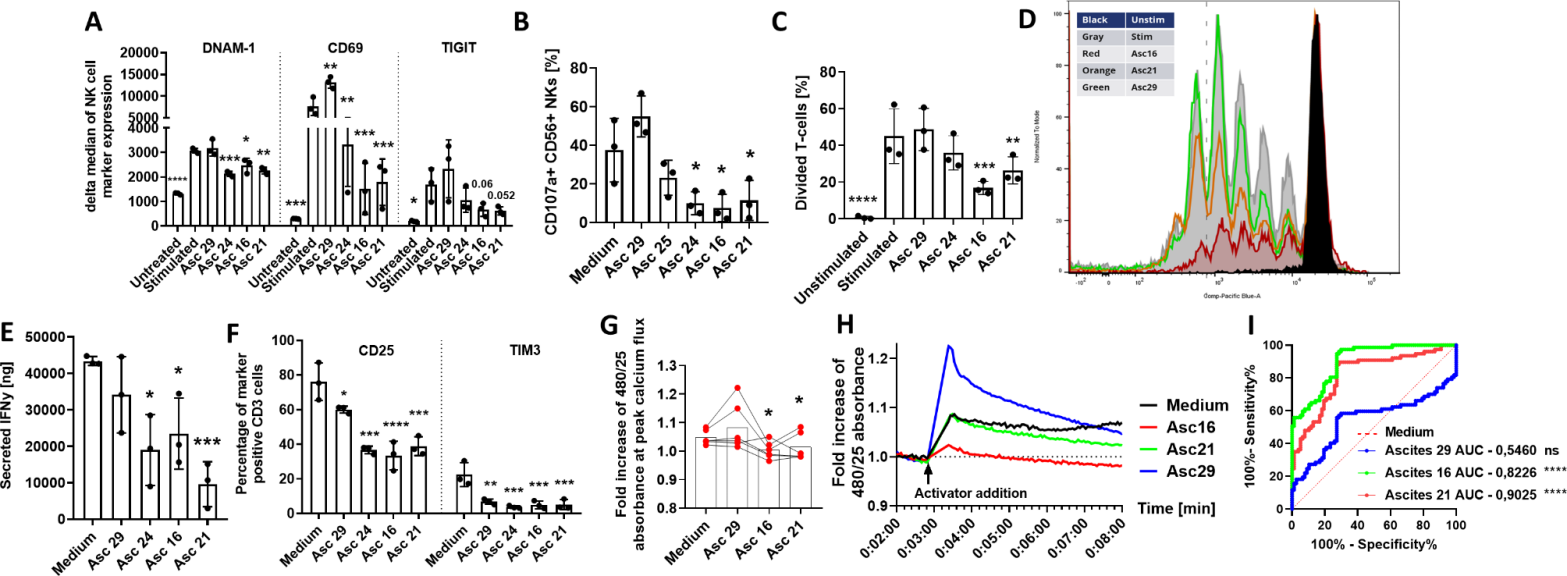
Malignant ascites causes dysfunction during in vitro activation of NK and T cells. **(A)** Expression of surface markers on stimulated NK cells in presence of ascites. Isolated healthy donor NK cells were coincubated with IL2 (400 U/ml) either in medium or 25%ascites-supplemented media. After 48 h expression of DNAM-1, CD69 and TIGIT was measured by FACS. **(B)** NK cytotoxicity of stimulated NK cells in presence of ascites. Percentage of CD107a-positive NK cells was assessed by FACS after 6-hours of stimulation with PMA (50ng/ml) and Ionomycin (1 µg/ml) either in media or 25% ascites-supplemented media. **(C-F)** Stimulation of T cells in presence of ascites. Isolated healthy donor T-cells were prestained with Cell Proliferation Dye EFluor 450 and stimulated using CD2/CD3/CD28 activator complex (1:40) either in medium or 25%ascites-supplemented media for four days. **(C)** Graph showing T cells proliferation. **(D)** Representative experiment showing T cell proliferation. **(E)** Secreted IFNγ [ng/ml] during T cell proliferation determined by ELISA. **(F)** Percentage of CD25-positive (left column) and TIM3-positive T cells (right column). **(G + H + I)** Intracellular Calcium-flux during T cell stimulation in presence of ascites. T cells were exposed to CD2/CD3/CD28 activator either in presence of ascites or medium. Intracellular Ca2 + flux was monitored via Fluo-4 dye. **(G)** Fold increase of 480/25 absorbance at peak of calcium flux. **(H)** Representative experiment of calcium flux after T cell stimulation in presence of ascites. **(I)** ROC analysis shows significant difference between calcium flux curves in suppressive ascites environment (red and green line) compared to medium (dotted red line). Data are presented as individual values with mean value as center of error bar ± standard deviation. Each datapoint represents one healthy donor. The normalization was done according to normal medium control. For significance testing ordinary one-way (2.A-C, E-F) and paired (2.G) ANOVA or ROC analysis (2.I) were performed, followed by Dunnett’s multiple comparison posthoc test between ascites and medium control group where appropriate. * (*p* < 0.05), ** (*p* < 0.01), *** (*p* < 0.001), **** (*p* < 0.0001)

After examination of ascites-mediated suppression of NK cells during their interaction with target cells, we next studied ascites-mediated inhibition in a target cell-independent system. To this end, we stimulated NK cells with IL2 in presence of ascites and determined the expression of the regulatory and activation surface markers. As illustrated in Fig. 2A the IL2-mediated upregulation of DNAM-1, CD69 and TIGIT was impaired by malignant ascites but not by benign sample. The expression of NKG2D and NKp46 was similarly affected, although to a lesser extent (data not shown). Comparably, malignant ascites also inhibited target-independent NK degranulation in PMA/Ionomycin-stimulated NK cells (Fig. 2B). To test whether ascites-mediated immunosuppression also affected T cells, we stimulated prestained T cells in the presence of ascites samples. As illustrated in Fig. 2C and D proliferation of T cells was significantly inhibited by malignant ascites. In parallel, IFNγ secretion of activated T cells was reduced (Fig. 2E), along with impaired upregulation of IL2-receptor alpha chain CD25 and activation/exhaustion marker TIM3 (Fig. 2F). Since the calcium influx into the cell is an important prerequisite for the early activation of central effector functions in immune cells, we studied calcium influx in activated T cells in the presence of ascites. We could demonstrate that calcium influx, particularly the peak of calcium influx, was substantially reduced upon addition of malignant suppressive ascites (Fig. 2G). In addition, this inhibition could be detected very early (2–3 min) after addition of the activating complex (Fig. 2H). Interestingly, benign ascites (no. 29) seemed to even support calcium flux into the cell. When assessed via ROC curve analysis, T-cell flux in medium was significantly different when compared to flux in suppressive ascites 16 and 21, while no significance was found for non-suppressive ascites 29 (Fig. 2I). We also tested IL2-stimulated NK cells against additional EGFR-positive cancer cells (A549) in presence of ascites and found that malignant ascites suppressed natural cytotoxicity and ADCC (Fig. S3B, Additional File 1.), tumor lysis (Fig. S3C, Additional File 1.) and conjugation (Fig. S3D, Additional File 1.) of IL2 stimulated NK cells as well.

In summary, here we could show that malignant ascites directly impairs immune cell functions, even in the absence of target cells. This suppression was observed for resting NK cells, stimulated NK cells and T cells.

### Electrolyte imbalance in malignant ascites is a major inhibitory mechanism during immunosuppression

**Fig. 3:**
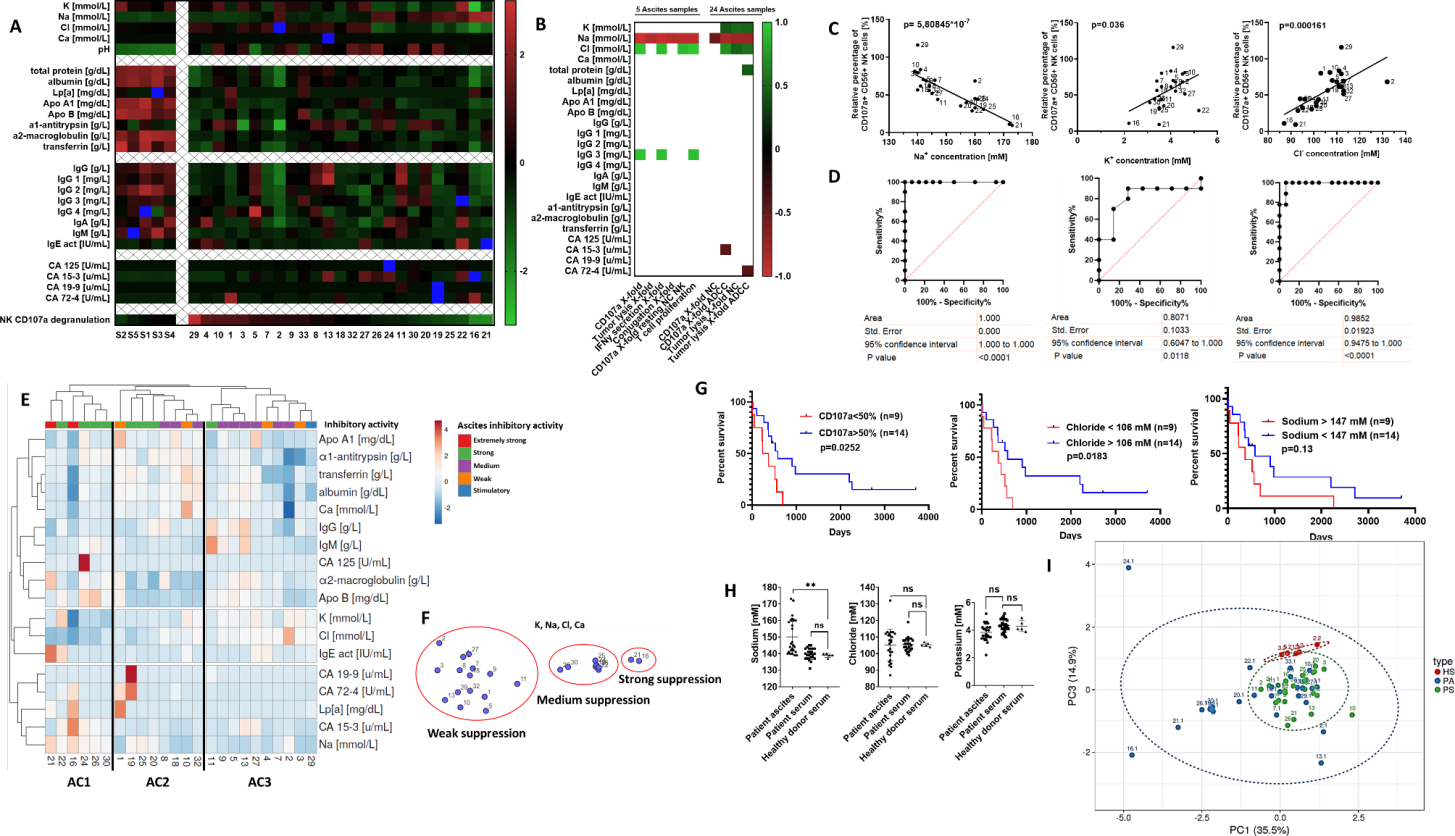
Aberrant electrolyte concentrations in malignant ascites contribute to suppression of immune cell functions. **(A)** Content of ascites and healthy donor serum. Components of ascites samples and healthy serum samples were determined by clinical chemistry analysis. Presented in the heatmap are calculated z-score values for each component. **(B)** Correlation between quantified ascites components and NK effector function. The matrix depicts significant positive and negative correlations (p < 0,05) between quantified ascites components and different NK effector functions. **(C)** Correlation between NK ADCC and electrolyte content in ascites. Pearson correlation shows negative correlation for sodium (left) and positive correlation for potassium (middle) and chloride (right) with NK ADCC. **(D)** Relationship between electrolytes in ascites and NK cytotoxicity. ROC (Receiver operating characteristic) curve showing the relationship between the concentrations of sodium (left), potassium (middle) and chloride (right) and 50% of NK cell degranulation inhibition. **(E and F)** Clustering of ascites samples. **(E)** Hierarchical clustering of ascites samples according to clinical chemistry data. **(F)** t-SNE plot showing the clustering of ascites samples according to electrolyte content. **(G)** Impact of electrolyte content in ascites on patient survival. Kaplan-Meier curves showing significant negative association of suppressed NK cytotoxicity (left), low chloride (middle) and high sodium content in ascites with patient survival. **(H and I)** Composition comparison of ascites, patient serum and healthy donor serum. **(H)** Comparison of the concentrations of sodium (left), chloride (medium), and potassium (right) between healthy donor and patient serum and ascites. **(I)** PCA plot showing clustering of ascites, patient and healthy donor serum done using clinical chemistry data. Two-tailed Pearson correlation (3.C) and ROC analysis (3.D) were used. Kaplan Meier curves (3.G) for overall survival were used with Mantel-Cox test (log-rank). Each datapoint represents one ascites sample. For significance testing ordinary one-way ANOVA was performed, followed by Dunnett’s multiple comparison posthoc test between ascites and healthy or patient serum (3.H). ns (non-significant), ** (p < 0.01)

In the next series of experiments, we aimed to identify components and molecular determinants which might be responsible for ascites-mediated immunosuppression. In an exploratory approach, we quantified various serum proteins and different electrolytes in ascites samples, corresponding patient serum as well as healthy donor serum and correlated the values to different NK effector functions (Fig. 3A, B). Interestingly, correlative statistical analysis revealed a potential connection between NK cytotoxicity and concentrations of distinct electrolytes (particularly, Na, K, Cl, Fig. 3B) in ascites. In detail, high concentration of sodium in ascites was in fact negatively correlated to NK ADCC while high concentrations of chloride and potassium showed positive correlation to cytotoxic activity of NK cells (Fig. 3B, C). Furthermore, by ROC analysis the electrolyte concentration served as a prognostic factor for a 50% reduction in NK activity (Fig. 3D). Hierarchical clustering (Fig. 3E) was performed using all obtained clinical chemistry data (taken from Fig. 3A). Ascites cluster 1 (AC1) contained samples with high sodium and low chloride content. All cluster 1 samples also had strong or very strong inhibitory activity (categories taken from table S3). In contrast, cluster 3 (AC3) displayed a different clinical chemistry profile and mostly contained ascites samples of medium or weak inhibitory activity. Thus, the three formed sample clusters mirror our ranked list of samples, separating them into strong, medium and weak suppressive clusters (Supplementary Table S3 (Additional File 1.), Fig S1A). To further substantiate the connection between electrolyte content and immunosuppressive activity of ascites we used the t-SNE method with ascites electrolyte data as dimensions (Fig. 3F). t-SNE plots grouped ascites samples into distinct clusters, which closely resembled the functional subgroups found in NK assays (Fig. 3C, left). Importantly, high chloride content in malignant ascites as well as the capacity of the ascites to reduce NK cytotoxicity were all correlated with poor survival of ascites-donors (Fig. 3G), which underscores a potential clinical relevance of our experimental findings. The investigated electrolyte imbalance was only specific to the ascites, as sodium concentration in matched patient peripheral blood sera did not differ from normal values in healthy donor control serum (Fig. 3H). A strong trend regarding chloride concentration in ascites was noted as well. Except for CA125 concentrations, no correlations could be found between patient serum and patient ascites components. Additionally, the concentration of patient serum components was not correlated to effector function (data not shown). To further assess differences between healthy donor serum, patient serum and ascites we performed PCA analysis using all clinical chemistry data, and found that a considerable number of patient ascites samples segregates from serum samples of patients and healthy donors (Fig. 3I).

**Fig. 4:**
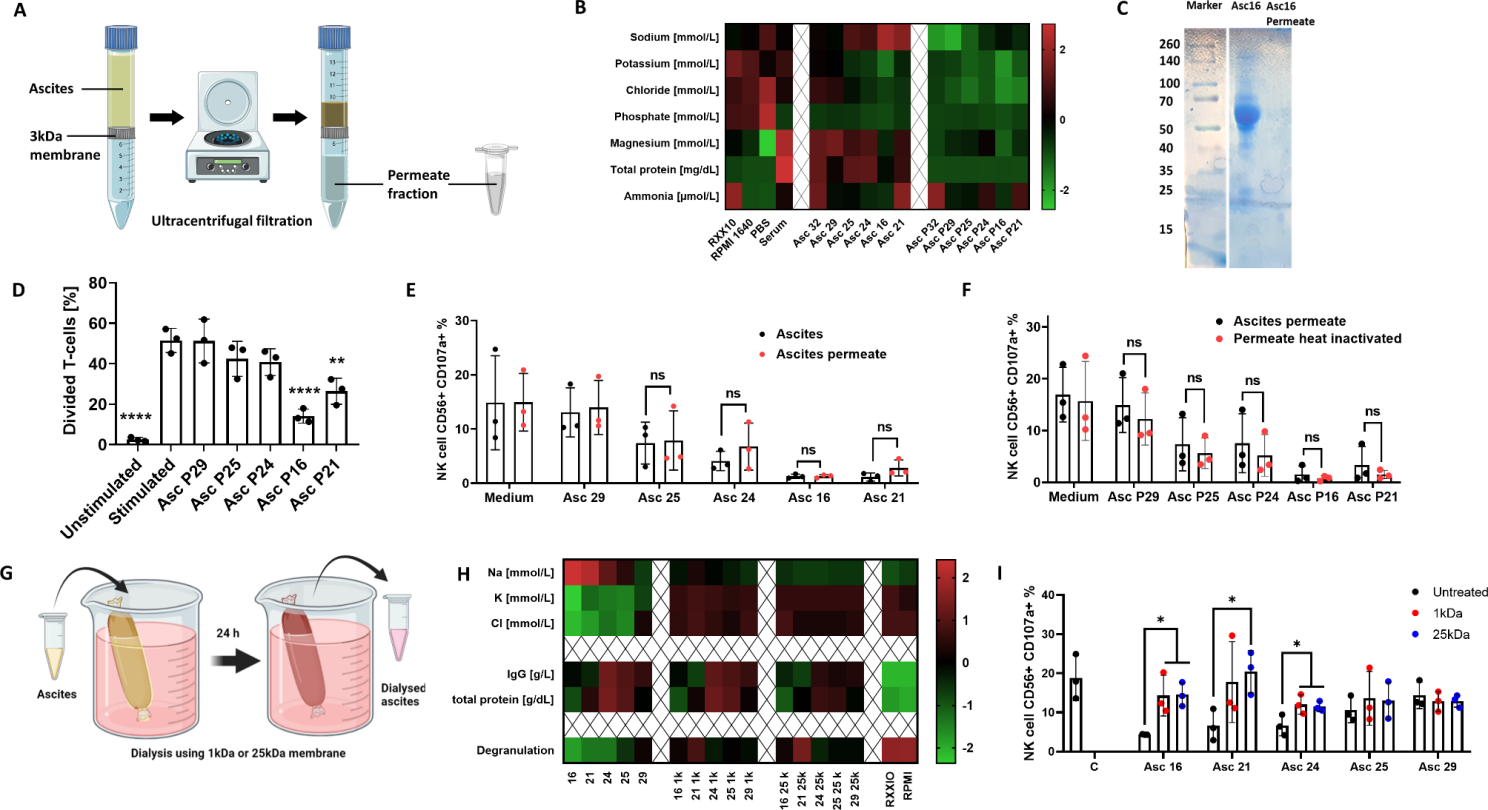
Ascites dialysis partially reverses inhibitory properties caused by non-protein and heat resistant components. **(A)** Graphical illustration of protein depletion in ascites. Ascites sample was processed via ultracentrifugal filtration and protein-rich retentate and protein-less permeate are collected. **(B)** Composition of ascites before and after protein depletion. Heatmap showing altered composition of protein-depleted ascites samples and protein-rich retentates. **(C)** Electrophoresis of ascites sample. Representative electrophoresis blot of ascites sample 16 and corresponding permeate. **(D)** Proliferation of activated T cells in presence of protein-depleted ascites. Prestained donor T-cells were stimulated using CD2/CD3/CD28 activator complex (1:40) in protein-less ascites permeate. **(E)** NK ADCC in presence of ascites permeate. Resting NK cells were coincubated in 1:1 ratio with IGROV1 cells with addition of ADCC-inducing anti-EGFR antibody Cetuximab in presence of unmodified or protein-less ascites permeate. **(F)** NK ADCC in presence of heat inactivated ascites permeate. Resting NK cells were coincubated in 1:1 ratio with IGROV1-cells and ADCC-inducing anti-EGFR antibody Cetuximab with protein-depleted ascites permeate which was heat inactivated at 90 °C for 1 h. **(G)** Graphical illustration of ascites dialysis in cell culture medium. Ascites samples were processed in medium overnight using 1 kDa or 25 kDa cutoff dialysis columns. **(H)** Composition of ascites before and after dialysis. Heatmap showing altered composition of ascites samples processed by dialysis. **(I)** NK ADCC in presence of dialyzed ascites. Resting NK cells were coincubated in 1:1 ratio with IGROV1 cells with addition of ADCC-inducing anti-EGFR antibody Cetuximab in presence of unmodified or dialyzed ascites. After 6 h expression of CD107a on NK cells was determined by flow cytometry. Each datapoint represents one healthy donor. For significance testing ordinary one-way ANOVA followed by Dunnet posthoc test (4.D) and two-way ANOVA followed by Sidak posthoc test was used (4.E, F and I). ns (non-significant), * (*p* < 0.05), ** (*p* < 0.01), **** (*p* < 0.0001)

Based on these detailed analyses of patient ascites, the consecutive experiments aimed to provide experimental evidence for an involvement of electrolyte imbalance in suppression of NK cytotoxicity. For this purpose, proteins in ascites samples were depleted using 3 kDa ultracentrifugation filters (Fig. 4A). Electrolyte and protein content was quantified in ascites permeate fraction (contains no proteins > 3 kDa) and ascites retentate fraction (all proteins > 3 kDa) by clinical chemistry analysis (Fig. 4B) and electrophoresis (Fig. 4C). With this approach we demonstrate that the ascites permeate fraction retained its capability to impair T cell proliferation and NK ADCC comparably to unmodified ascites (Fig. 4D and E). Furthermore, the inhibitory effect of the permeate fraction was still present after heat treatment at 90 °C indicating that the inhibitory component is heat-resistant (Fig. 4F). The inhibitory effect persisted even after performing charcoal stripping, which excludes lipids and other lipid-based molecules as main inhibitory mediators (Fig. S4A and B). To further explore the impact of these small heat resistant non-lipid molecules, we performed dialysis for 24 h on ascites samples using 1 kDa or 25 kDa dialysis columns (Fig. 4G). After the dialysis, electrolyte and protein content were quantified and it was shown that electrolyte concentrations were successfully restored to levels contained in cell culture medium RPMI 1640, in which dialysis was performed (Fig. 4H). Additionally, no substantial loss of proteins or IgG antibodies happened during the dialysis. When dialyzed samples were used in NK degranulation assay, NK cells achieved significantly higher degranulation compared to untreated samples (Fig. 4I). The restorative effect was the strongest in ascites samples with highest electrolyte imbalance.

All presented data support the hypothesis that electrolyte content, and not protein function, inhibits antitumoral NK and T cell activity in malignant ascites. Specifically, high concentrations of sodium and low content of chloride and potassium caused reduced effector function.

### Malignant ascites alters expression of ion channels, signaling pathways and effector molecules

**Fig. 5:**
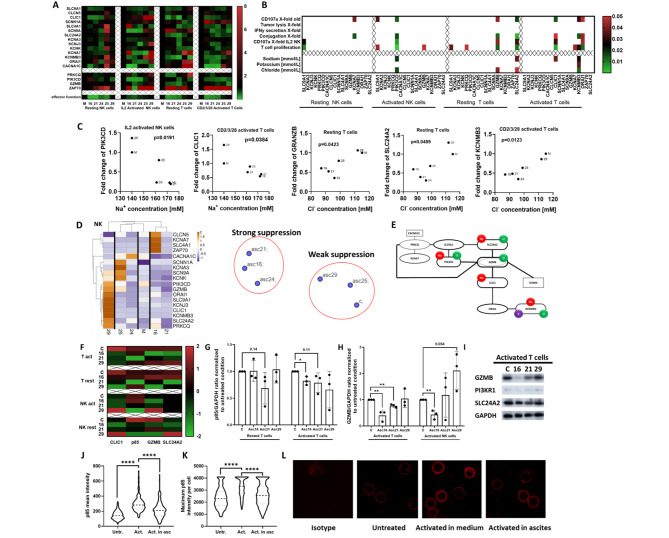
Malignant ascites causes transcriptional and translational changes of signaling proteins and electrolyte channels in immune cells. **(A)** Heatmap showing altered transcriptional gene expression in immune cells in presence of ascites. Resting NK cells were incubated with ascites for 6 h, and IL2 activated NK cells for 24 h. Resting T cells were incubated with ascites for 24 h and the activation complex was treated for 48 h. Total RNA was isolated from NK and T cells. Quantitative real-time PCR was conducted with annealing temperature of 62 °C for all primers. Primers are listed in Supplementary Table S2**(B)** Correlation between NK effector function, electrolyte content in ascites and altered mRNA-expression of selected genes. Correlation matrix depicts significant gene expression changes (p < 0,05) in resting and activating NK cells (left) and resting and activated T cells (right). **(C)** Correlation between electrolyte content in ascites and altered mRNA-expressions of selected genes. Significant Pearson correlations of gene expression changes and electrolyte content in ascites. Sodium correlation to PI3K in IL2-activated NK cells (first panel). Chloride correlation to Granzyme B (third panel) and SLC24A2 (fourth panel) in resting T cells. Correlation of CLIC1 to sodium (second panel) and KCNMB3 to chloride (fifth panel) in complex activated T cells. **(D)** Clustering of mRNA-transcripts of stimulated NK cells in presence of ascites. Hierarchical clustering done according to gene expression data from ascites treated activated NK cells (left). t-SNE plot demonstrating unbiased grouping of ascites samples according to most affected genes (right). **(E)** Graphical illustration of crucial transcriptional changes in NK and T cells after ascites exposure. Portrayed genes were affected by ascites exposure significantly as shown by both ANOVA and Pearson correlation. Lines connecting genes show Pearson correlation relationships. Every gene affected by specific electrolyte was marked with appropriate symbol (Na-red, Cl-Green and K-Purple). **(F, G, H and I)** Protein expression of electrolyte channels and immune effector molecules in NK and T cells affected by ascites. Both NK and T cells were incubated in medium or 25% ascites supplemented medium for 24 h (resting cells) or 48 h (activated cells). NK cells were treated with 50 ng/ml of PMA, 1 µg/ml of Ionomycin, while T cells were activated by adding 5 µl of CD2/CD3/CD28 activator. Protein expression was determined by densitometric analysis of western-blots and normalized to GAPDH. **(F)** Heatmap overview showing protein expression comparison between the healthy donor (C), two inhibitory (No.16, 21), and one non-inhibitory (No.29) ascites- treated NK and T cell samples. **(G)** p85 expression in samples from resting and activated T cells as determined by western blot. **(H)** Bar graph depicting different Granzyme B expression in samples from activated T and NK cells. **(I)** Western blot bands from representative experiment. **(J, K and L)** Ascites-mediated inhibition of p85 membrane recruitment in T cells. Isolated T cells were activated for 5 min with CD2/CD3/CD28 activator in either medium or ascites 21 supplemented medium. Surface localization and expression of p85 was determined by confocal microscopy. **(J)** Violin plot depicting p85 mean intensity on T cell surface. **(K)** Violin plot depicting the maximum of p85 intensity of each cell. **(L)** Representative confocal microscopy images showing T cell surface expression of p85 (red) in different conditions. Data are presented as individual values with mean value as center of error bar ± standard deviation. For significance testing two-tailed Pearson correlation were used (5.B, C), paired t-test was used to assess the significance (5. G, H), ordinary one-way ANOVA and Dunnett posthoc (5.J. K). *(*p* < 0.05), **(*p* < 0.01), ****(*p* < 0.0001)

In the next series of experiments, we wanted to identify ion channels and down-stream signaling pathways that were modulated by our clinical ascites samples. To this end, resting and IL2-stimulated NK cells as well as T cells were exposed to different ascites samples. In an explorative analysis we assessed the mRNA-expression of 18 different highly expressed genes which are known to be crucial [21] for signal transduction during various effector functions of NK and T cells and examined expression levels of essential electrolyte channels (Fig. 5A). Further statistical analysis revealed significant correlations between immune cell effector function, ascites electrolyte composition and mRNA-expression of selected genes (Fig. 5B). Suppressive ascites samples induced significant downregulation of signaling molecules such as PIK3CD (Fig. S5A, left, Additional File 1.) and PRKCQ (Fig. S5B, left, Additional File 1.). As expected, expression of these signaling molecules correlated with effector functions in NK (Fig. S5A, right, Additional File 1.) and T cells (Fig. S5B, left, Additional File 1.). In the same way, ascites affected transcript levels of various ion channels, for example, SLC24A2 (NCKX4, Sodium/potassium/calcium exchanger 4), which was found to have a strong positive correlation to most effector functions (Fig S5B, right, Additional File 1.). We also observed significantly reduced expression of CLIC1 (chloride intracellular channel protein 1) in presence of malignant ascites (Fig. S5C, right, Additional File 1.) and CLIC1 expression was connected to NK ADCC degranulation (Fig.S5C, left, Additional File 1.). Finally, we wanted to link those findings to actual electrolyte concentrations in ascites samples. To achieve this, we plotted concentrations of sodium and chloride against expression levels of molecules from our gene expression analysis and found a significant correlation between the content of sodium and chloride and expression level changes of PIK3CD, CLIC1, GRANZB, SLC24A2, and KCNMB3 respectively (Fig. 5C). Considering all significant gene expression changes and correlations, we aimed to see if assessed genes could serve as a specific signature of ascites-induced changes. Using hierarchical clustering on gene expression data of activated NK cells (Fig. 5D, left) we were able to generate heatmaps that successfully clustered treated cells depending on which ascites samples they were exposed to. Similarly, by using gene expression data from IL2-activated NK cell for t-SNE, ascites samples segregated into more and less suppressive groups (Fig. 5D, right). Our data suggest a functional connection between electrolyte imbalance, ion channels and immune effector function, which we have portrayed in a gene network map that summarizes the connections between the individual genes and the impact of different electrolytes on their expression (Fig. 5E).

To confirm the clinical relevance of our data we assessed the expression of investigated genes in T cells isolated from ascites of two ovarian cancer patients. The expressions of PIK3CD, AKT1, SLC9A1, and SCNN1A transcripts were consistently downregulated in both patient ascites-isolated T cells and two ascites-treated healthy donor T cells (Fig. S6A). Additionally, ZAP70, SCN9A, and CLCN5 were upregulated in patient cells similar to one of the ascites treated samples (Fig. S6B). T cells from ascites also showed a unique gene set that was not induced in HD T cells exposed to ascites (e.g. PRKCQ, GZMB, CACNA1C) (Fig. S6A, left). To further demonstrate the similarity between ascites-isolated and ascites-treated T cells we used the t-SNE method with gene expression data as dimensions (Fig. S6C). t-SNE plot grouped untreated healthy donor T cells in distance to ascites-treated and patient-isolated T cells. In addition, we performed a re-analysis of published RNAseq dataset by Fraser et al. [21] and found that GZMB, SLC4A1 and CACNAC1 and KCNA7 were similarly downregulated in both ascites-treated and patient-isolated NK cells (Fraser study, data not shown) and our study (Fig. 5A).

For further confirmation, we also assessed the protein expression of selected important ion channels and effector molecules to show that ascites-induced effects are not limited to the level of transcript. As illustrated in Fig. 5F ascites No.16 and No.21 negatively affected the expression of all investigated proteins in both T and NK cells in either rested or activated state (Fig. 5F). The most pronounced ascites-induced effects were the significant decreases in p85 and Granzyme B protein expressions, especially observed during T and NK cell activation (Fig. 5G - I).

Since PI3K plays a crucial role in T cell signalling and the membrane recruitment of p85 can be used for defining T cell activation state, our next experiments examined the p85 membrane expression in presence of ascites. Our first assessment confirmed that p85 is indeed localized on the membrane and is present to some degree even in resting non-activated T cells. As illustrated in Fig. 5J, K and L (second and third panel) the activation of T cells induced a strong increase of p85 membrane recruitment compared to untreated control cells. However, the presence of ascites during the activation significantly impaired p85 membrane expression (Fig. 5J, K and L (forth panel)). These data support the important role of PI3K in activation and ascites-mediated inhibition of functional T cells.

In summary, our experiments show that malignant ascites substantially downregulated the transcript and protein expression of various signal transduction molecules, notably PI3K, as well as distinct ion channels.

### Sodium channel inhibitors prevent ascites-induced sodium influx and immunosuppressive effects

**Fig. 6:**
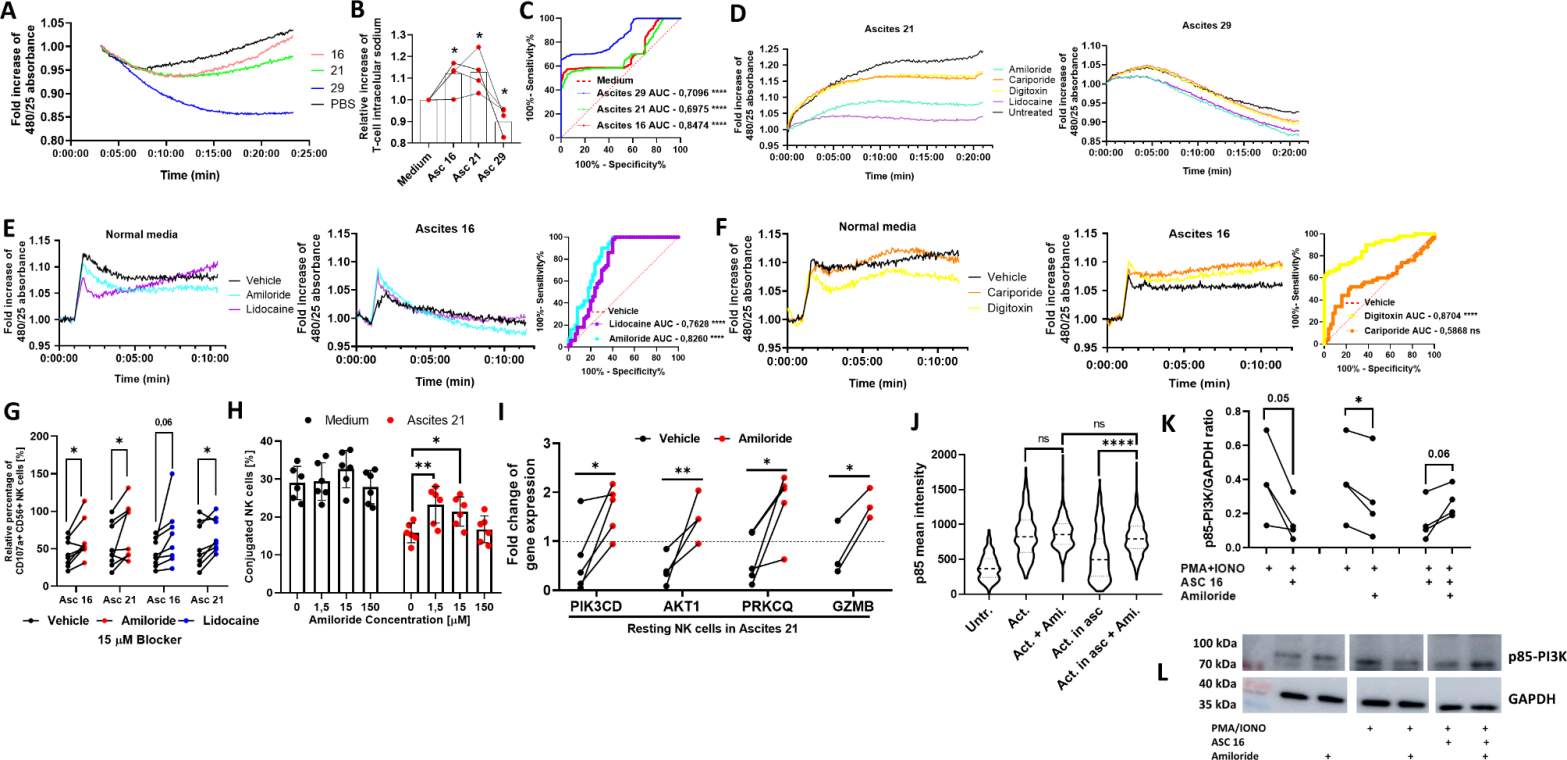
Malignant ascites-mediated immunosuppression can partially be reversed by inhibitors of sodium channels. **(A, B and C)** Sodium influx in T cells in presence of ascites. T cells were exposed to 25%-ascites supplemented media or PBS and media control, respectively. After 25 min. influx of sodium was measured using fluorescent CoroNa Green dye. **(A)** Representative experiment showing sodium influx in T cells in presence of different malignant ascites samples. **(B)** Relative increase of intracellular sodium in T cells in presence of different ascites samples. Comparison is made between experiment endpoint values. **(C)** ROC curve analysis shows significant difference between measured sodium flux curves in suppressive ascites environment (red and green line) compared to medium (dotted red line). **(D)** Sodium influx in T cells in presence of ascites and inhibitors of sodium channels. CoroNa Green prestained resting T-cells were exposed to different samples of 25%-ascites-supplemented media (left: ascites 21, right: ascites 29) and media control, respectively. Different inhibitors of sodium channels were added (150 µM). Results were normalized to starting fluorescence. Representative experiments are shown. **(E and F)** Calcium influx in activated T cells in presence of ascites and inhibitors of sodium channels Fluo-4 prestained resting T cells were activated using CD2/CD3/CD28 complex either in normal media or high sodium malignant ascites sample No.16. Sodium channel inhibitor were added with activator complex simultaneously. **(E)** Representative experiment of calcium influx in activated T cells in presence of amiloride and lidocaine and ascites 16 (middle panel) and media control (left panel). ROC curve analysis (right panel) shows significant differences between measured calcium flux curves in ascites 16 with addition of amiloride (cyan line) or lidocaine (purple line), compared to vehicle control (dotted red line). **(F)** Representative experiment of calcium influx in activated T cells in presence of cariporide and digitoxin and ascites 16 (middle panel) and media control (left panel). ROC curve analysis (right panel) shows significant difference between measured calcium flux curves in ascites 16 with addition of cariporide (orange line) or digitoxin (yellow line), compared to vehicle control (dotted red line). **(G)** NK cell degranulation in presence of ascites and sodium channel inhibitors. Isolated NK cells were pretreated with water (vehicle control) or 15 µM addition of amiloride or lidocaine for one hour. Washed NK cells were exposed to 25%-ascites supplemented media and media control, respectively. After 6 h NK cell degranulation was measured using FACS. **(H)** NK-TC conjugation in presence of ascites and amiloride. Isolated NK cells were pretreated with water (vehicle control) or 1,5, 15 or 150 µM addition of amiloride for one hour. NK cells and IGROV1-cells were mixed in 4:1 effector to target ratio in presence of 1 µg/ml Cetuximab and in presence or absence of 25%-ascites-supplemented media. **(I)** Expression of NK cell signal transduction and effector molecules in presence of ascites 21 and amiloride. Resting and activated NK cells were incubated with ascites sample No. 21 and water (vehicle control) or 150 µM addition of amiloride for 3 h. Fold change of PIK3CD, AKT1, PRKCQ and GZMB gene expressions. Gene expression was normalized to housekeeping gene and respective ascites treated controls. **(J)** p85 membrane recruitment in activated T cells in presence of ascites 21 and amiloride. Isolated T cells were activated with CD2/CD3/CD28 activator in either medium or ascites 21 supplemented medium with addition of 150 µM of Amiloride for five minutes. Surface localization and expression of p85 was determined by confocal microscopy. Violin plot depicts p85 mean intensity on T cell surface. **(K and L)** Phosphorylation of signal transduction protein p85-PI3K in NK cells in presence of ascites 16 and amiloride. **(K)** Isolated NK cells were exposed to were exposed to 25%-ascites supplemented media and media control. During the incubation, NK cells were treated with 50 ng/ml of PMA, 1 µg/ml of Ionomycin and/or 150 µM of amiloride for five minutes. Phosphorylation was determined by densitometry and normalized to GAPDH and respective ascites treated controls. **(L)** Western blot bands from representative experiment and GAPDH control. Each datapoint represents one healthy donor. For significance testing RM one-way ANOVA followed by Dunnett’s multiple comparison posthoc test (6.B and J), two-way ANOVA followed by Dunnett’s multiple comparison posthoc test (6.H) and paired t-test were used (6.G, I and K). ns (non-significant), * (*p* < 0.05), ** (*p* < 0.01), ****(p < 0.0001).

As our current data suggest that high concentrations of sodium in ascites are causally involved in suppression of immune cell function, in our final series of experiments we used selected inhibitors of sodium channels and examined their potential as modulators of ascites-induced immunosuppression. First, we determined the intracellular concentration of sodium in T cells upon coincubation with different ascites samples. As illustrated in Fig. 6A and B high sodium ascites samples (No. 16 and 21) induced a significant influx of sodium during the 25-minute period of incubation, while the low sodium benign ascites sample No. 29 caused a significant efflux of intracellular sodium (Fig. 6A, B and C). Interestingly, PBS control also induced influx of sodium (Fig. 6A) which may be due to the low chloride-high sodium content in PBS solution. Next, we used the sodium channels inhibitors amiloride, lidocaine, cariporide and digitoxin in order to manipulate the sodium flux in T cells in presence of different ascites samples. We found that all used inhibitors (Fig. 6D, left) prevented sodium influx in T cells in presence of high sodium ascites sample (No. 21). In contrast, in presence of benign ascites (No. 29), which has physiological sodium content, the influx of sodium was only minimally altered. On the contrary, particularly amiloride and lidocaine were even able to augment efflux of sodium into extracellular space (Fig. 6D, right).

As we could demonstrate that malignant ascites is able to impair calcium influx into T cells (Fig. 2G and H), which subsequently suppressed immune cell functions, we examined whether sodium channel inhibitors were able to normalize calcium flux in activated T cells. Indeed, most of the tested sodium channel inhibitors restored calcium influx in activated T cells incubated with high sodium ascites No. 16 (Fig. 6E and F, middle panels). Restorative effects of amiloride, lidocaine and digitoxin on T cell calcium flux in ascites environment were confirmed as significant by ROC curve analysis (Fig. 6E and F, right panels). The preincubation of NK cells with amiloride or lidocaine prior to ADCC assay in ascites environment partially restored NK degranulation (Fig. 6G). Similarly, preincubating NK cells with amiloride significantly reversed ascites-induced inhibition of NK conjugation (Fig. 6H).

In order to further substantiate these data, we examined the expression of different signaling molecules in NK cells in presence of malignant ascites (no.21) and sodium channel inhibitors. Here we observed that sodium blocker amiloride restored ascites-induced downregulation of PIK3CD, PRKCQ, GZMB and AKT1 in resting NK cells incubated with ascites (Fig. 6I). Lidocaine had a similar, but less pronounced effect in activated cells (data not shown). In line with these data the addition of amiloride during T cell activation also restored membrane recruitment of p85 in ascites treated cells (Fig. 6J). Lastly, western blot was performed to examine the impact of ascites and amiloride on phosphorylation of p85-PI3K regulatory subunit in PMA/Ionomycin activated NK cells. Here we were able to demonstrate that after five minutes PI3K phosphorylation was impaired by presence of ascites. Interestingly, while amiloride caused a moderate inhibition of PI3K phosphorylation in medium conditions, in presence of sodium rich ascites the inhibitor increased phosphorylation and this counteracted ascites-induced suppression in all four independently performed experiments (Fig. 6K and L).

**Fig. 7:**
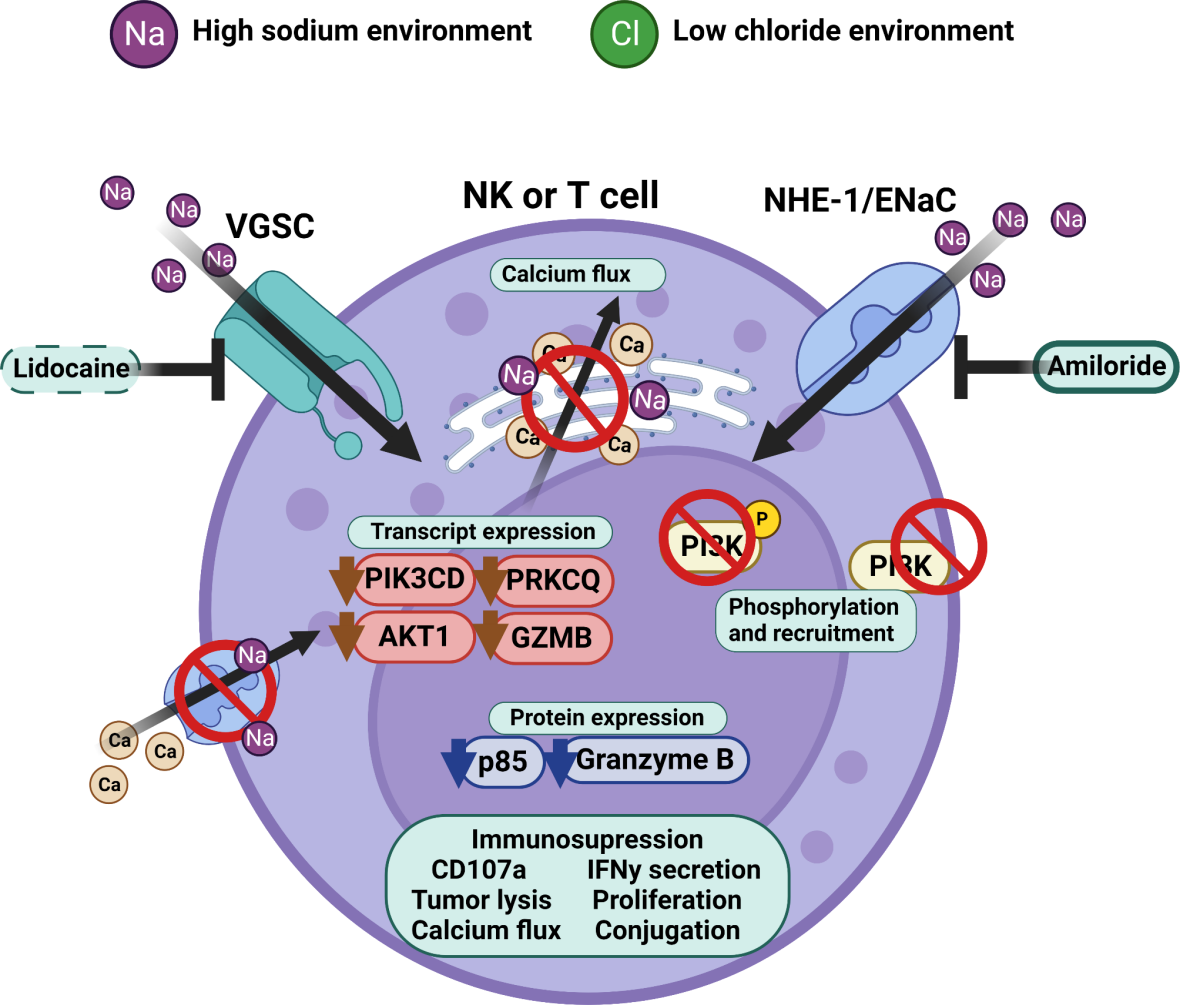
Illustration of proposed inhibitory molecular mechanism. Malignant peritoneal ascites is characterized by a sodium/chloride imbalance. This imbalance suppresses NK and T cell effector function by interfering with calcium signaling and signal transduction. Sodium channel blockers can prevent inhibitory effects caused by excess of sodium. Illustration was created with BioRender

In summary, the presented data show that a sodium imbalance in ascites is mechanistically involved in regulation of calcium flux, downstream signaling, and NK/T effector functions (Fig. 7).

## Discussion

Immunotherapeutic approaches offer great potential for cancer treatment, but success in epithelial ovarian cancer is still limited. Advanced or recurrent ovarian cancer is mostly associated with the presence of malignant ascites, a pathological fluid accumulation in the peritoneal cavity [6]. Its heterogeneous composition generates a unique tumor microenvironment that has long been known to mediate immunosuppression and promote disease progression [8]. Thus, malignant ascites limits response to immunotherapy and results in poor patient survival [7].

At present, the molecular mechanisms that mediate the immunosuppressive effects of malignant ascites are still poorly understood. Previous studies focused on the characterization of cellular components and studied tumor cell-related mechanisms promoting tumor growth and metastasis [15, 25]. Further studies regarding soluble factors mainly concentrated on analyzing cytokines or metabolites and for some of those factors, prognostic relevance or immunosuppressive effects could be demonstrated [17, 18]. Furthermore, ascites protein fraction might contribute to immunosuppression, since even some healthy serum proteins are reported to negatively regulate immune cell activity [26, 27]. Among these, intrinsic serum immunoglobulins are able to compete with therapeutic antibody and impair ADCC [28]. However, existing reports about properties of potential inhibitory factors in malignant ascites are inconsistent regarding their immunosuppressive power, sensitivity to heat, or degradation by proteases [20, 29, 30]. These inconsistencies could be based on methodological challenges as the application of standard biochemical methods, precipitation agents [20, 30, 31], proteases in different vehicle fluids [20, 29, 30] and other chemical additives [29, 32] might result in irreversible alteration of the original ascites. Other studies used methods of chromatographic fractionalization with the potential risk of dilution or loss of proteins and other non-proteinous components [20]. Thus, methodological challenges in biochemically separating ascites components, while retaining full biological activity, could have contributed to the failure of fully identifying immunosuppressive components in ascites so far.

In the first part of our study, we examined the immunosuppressive potential of malignant ascites on antitumoral activity of NK cells. We compared different ascites samples of patients with advanced or recurrent ovarian cancer, gastrointestinal adenocarcinoma and benign diagnosis, respectively. We studied their impact on different effector functions of NK cells directed to ovarian cancer cells. We could demonstrate that cytotoxic and secretory NK cell functions were substantially impaired by malignant ascites which is consistent with other studies [16]. Upregulation of NK activation marker CD69 and TIGIT as well as downregulation of DNAM-1 was inhibited, suggesting that ascites-mediated immunosuppression was mainly due to hindered NK cell activation and not due to NK cell exhaustion [16]. In line with some previously published work, we showed that inhibition was independent from presence of target cells and also affected T cell activity directly [33, 34]. Of note, also downregulation of HLA-E, a molecule implicated in the regulation NK activity under certain conditions, did not affect tumor cell lysis in our systems. Interestingly, studying flux of calcium ions in proliferative T cells in presence of ascites demonstrated that inhibition of effector cell activity was initiated early, after few minutes. Accordingly, NK cell killing and conjugation was already affected shortly after incubation with ascites. Remarkably, all suppressive effects were only mediated by malignant ascites but not by benign sample derived from patient with congestive heart failure.

In the second part of our study, we aimed to identify novel factors and mechanisms which are responsible for immunosuppression in malignant ascites. In contrast to previous qualitative approaches [20, 30], we initially performed a comprehensive quantitative analysis of ascites composition. To this end, we fractionated acellular ascites samples without chemical alteration. With this approach, we found that a small, non-proteinous component was responsible for immunosuppressive activity. Thus, we quantified the concentrations of various electrolytes and serum proteins in ascites samples by clinical chemistry analysis and correlated our results to antitumoral NK cell activity and patient survival. Interestingly, we could identify aberrant electrolyte content in malignant ascites as novel factors suppressing immune cell functions. Specifically, high sodium content as well as low content of chloride and potassium were significantly correlated with reduced NK and T cell effector function. Thereby, ROC curve analysis revealed that all three electrolytes predicted inhibition of immune functions. Furthermore, high sodium was significantly correlated to patient poor survival in our cohort. In contrast, excessive chloride content was significantly associated with favorable patient outcome suggesting an independent protecting role by positive modulation of immune function. In addition, the extent of NK inhibition by malignant ascites was correlated to patient outcome as well. In conclusion, the association between NK inhibition and patient survival also suggests that the degree of NK inhibition corresponds to immunosuppression in the peritoneal cavity of patients. Further reprocessing of ascites samples revealed that after ultracentrifugation protein-depleted permeate fraction still suppressed NK cell cytotoxicity and T cell proliferation. Additionally, by inactivating ascites samples by heating, we confirmed that potential inhibitors are heat resistant. Charcoal stripping excluded lipids as major mediators of suppression. Correction of the electrolyte imbalance by dialysis abrogated the immunosuppressive effect, especially in ascites samples with strong electrolyte imbalance. The hypothesis of electrolyte-mediated suppression of immune cell functions has been explored in some earlier studies [35–37]. These studies provided initial evidence that sodium and potassium cations may affect NK cell function. It was reported that NK cell function was reduced in environments with unphysiologically low or high concentrations of sodium (75nM – 150 nM) [38]. Similarly, monocyte-mediated ADCC was affected by extracellular Na^+^ and K^+^ concentrations [35]. However, these studies were mostly observational and often no detailed underlying molecular mechanisms could be identified. More recent studies predominantly focused on the impact of high salt diet on immune cell functions [39, 40] and the relevance of high sodium in the microenvironment of solid tumors [41]. In this context, extracellular potassium originating from necrotic tumor areas could impair T cell effector functions [42]. A possible explanation for suppression in these examples is that ions can bind to protein helices, which changes their conformation and can prevent interaction with ligands or other proteins [43]. Two studies show that chloride binding to specific residues affected the catalytic activity of pancreatic α-amylase [44] and the permeability of the SLC4A1 channel [45]. Beside regulating enzymatic activities in phagocytes [46], in the few existing studies chloride was shown to be essential for regulating immune cell function [47, 48]. In conjunction with our findings, these data from other disease settings underscore the relevance of electrolytes homeostasis and imbalance for proper immune function or dysfunction, respectively.

In the final part of our study, we mechanistically explored a novel immunosuppressive mechanism in the liquid tumor microenvironment of the peritoneal cavity. This mechanism is primarily mediated by defective intracellular calcium signaling as well as modulation of signal transduction pathways and ion channels of NK and T cells. We confirmed that specific ion channels such as SLC9A1 (NHE-1), SLC24A2 (NCKX4), and SCNA9 (Nav1.7, VGSC) were closely linked to other key effector molecules like Granzyme B or signal transducers like PI3K-δ and PKC-θ, which are necessary for proper immune function. Furthermore, malignant ascites induced downregulation of Granzyme B and PI3K-δ not only on transcription level, but also on protein level, which correlated to weaker effector function and ascites sodium content. In addition, the presence of inhibitory ascites impaired the rapid membrane recruitment of p85 (the 85KDa regulatory subunit of PI3Kinase) during the T cell activation, which provides a molecular basis for the early onset of activation failure.

To provide causal evidence for the involvement of ion channels, we used the channel blocker amiloride (ENaC/NHE-1), lidocaine (VGSC), cariporide (NHE-1), and digitoxin (Na+/K + ATPase) to modulate the activity of sodium channels. We observed immunosuppressive effects of sodium excess on T cell activation as sodium channel blockers amiloride, lidocaine and digitoxin restored T cell calcium influx in sodium-rich ascites coculture within first five minutes of exposure. In subsequent experiments preincubation with amiloride or lidocaine blockers had caused a reversal of ascites-mediated inhibition of effector functions. Both NK cell degranulation (six hours) and NK-TC conjugation (45 min) were significantly higher after amiloride or lidocaine pretreatment. Furthermore, using a similar approach we confirmed the mechanism of sodium-induced aberrant gene expression. The direct addition of sodium channel blocker, amiloride, prevented ascites-mediated downregulation of PIK3CD, PRKCQ, AKT1, and GZMB transcripts. After short activation and exposure to ascites environment (five minutes), amiloride was also able to partially restore the phosphorylation of signaling protein p85-PI3K and to completely restore its membrane recruitment in NK and T cells, respectively. These experiments open the possibility to use sodium channel blockers as immunomodulatory agents that may restore immune activity in suppressive microenvironments [36, 49].

## Conclusion

In summary, here we report novel factors and mechanisms causing immunosuppression in malignant ascites in peritoneal carcinomatosis. The quantitative analysis of various acellular components in malignant ascites and their correlation to impaired antitumoral NK activity identified imbalanced electrolytes (sodium, potassium and chloride) as the source of inhibition. High sodium content was proven to substantially inhibit all crucial effector functions of NK cells and impair T cell activity as well. Furthermore, we could show for the first time that the extent of immunosuppression on NK cytotoxicity is correlated to patient poor survival. Unexpectedly, the positive correlation of chloride content to patient outcome suggests a protective effect of elevated chloride. Therapeutic application of selected ion channel inhibitors may provide novel means to restore immune effector cell activity in ascites and counteract immunosuppression.

### Electronic supplementary material

Below is the link to the electronic supplementary material.

Supplementary Material 1

## Data Availability

The original contributions presented in the study are included in the article/Supplementary Material. Further inquiries about the data access can be directed to the corresponding author.

## Abbreviations

NC: Natural toxicity
ADCC: Antibody-dependent cellular cytotoxicity
ASC: Ascites
AC: Ascites cluster
P: Permeate
Act.: Activated
Untr.: Untreated
Ami.: Amiloride
