# Supplementary material for: Electrolyte imbalance causes suppression of NK and T cell effector function in malignant ascites
Source: J Exp Clin Cancer Res. 2023 Sep 8;42:235. doi: 10.1186/s13046-023-02798-8 (PMC10485936; doi:10.1186/s13046-023-02798-8)
Supplement: Supplementary file 1 — Supplementary Material 1 [file 13046_2023_2798_MOESM1_ESM.docx]

**
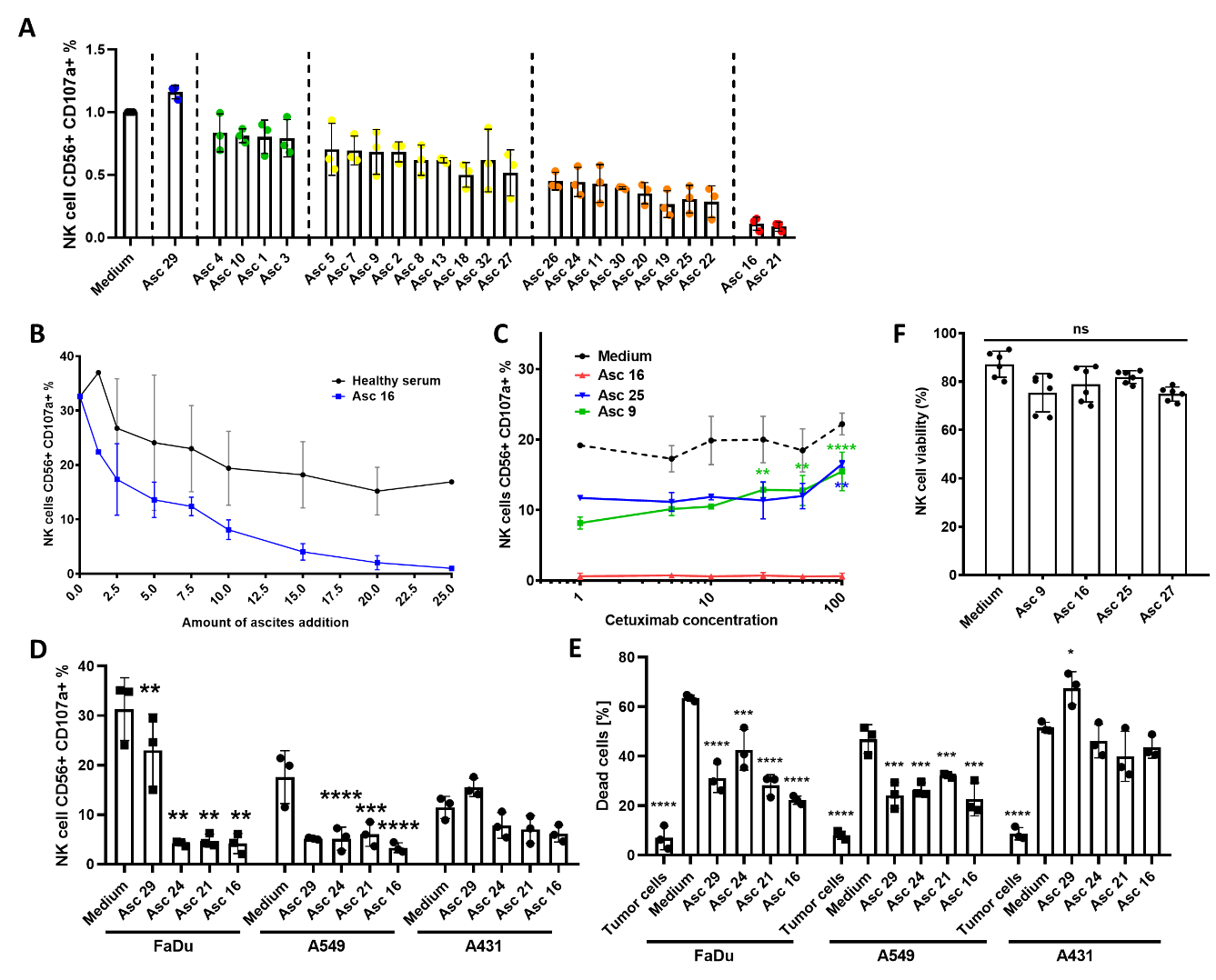
**

**Supplementary Figure 1. – Malignant ascites impairs interaction between NK cells and different types of EGFR-positive cancer cells. (A) Ascites dependent inhibition of NK-ADCC.** NK cells were coincubated in 1:1 ratio with EGFR-positive IGROV1 ovarian cancer cells in presence of various ascites samples and ADCC-inducing antibody Cetuximab (1 µg/ml). Percentage of CD107a-positive NK cells was determined after 6 hours by flow cytometry and was normalized to medium control. **(B) Comparison of NK-ADCC inhibition by malignant ascites and healthy donor serum.** NK cells were coincubated in 1:1 ratio with EGFR-positive A549 lung adenocarcinoma cells in increasing concentration of healthy donor serum or ascites 16 added to media and ADCC-inducing antibody Cetuximab (1 µg/ml). Percentage of CD107a-positive NK cells was determined after 6 hours by flow cytometry. **(C) Cetuximab-concentration-independent inhibition of NK-ADCC by malignant ascites.** NK cells were coincubated with A549 lung adenocarcinoma cells in 1:1 ratio either in media or media supplemented with 25% of different malignant ascites samples. ADCC-inducing antibody Cetuximab was added in increasing concentration. Percentage of CD107a-positive NK cells was measured after 6 hours by flow cytometry. **(D and E)** **NK ADCC and tumor lysis of EGFR-positive cancer cells in presence of ascites.** NK cells were coincubated in 1:1 ratio with head and neck cancer cell line FaDu, lung cancer cell line A549 and epidermoid cancer cells, respectively. Cetuximab (1 µg/ml) was added with or without 25% ascites supplemented media. **(C)** After 6 hours percentage of CD107a-positive NK cells directed against FaDu (left column), A549 (middle column) and A431 (right column) was determined by flow cytometry. **(D)** After 24 hours percentage of lysed FaDu cells (left column), A549 (middle column) and A431 cells (right column) was quantified by Annexin/7AAD-staining in the flow cytometer. **(F) NK cell viability in presence of ascites.** NK cells were coincubated with media or media supplemented with 25% of different ascites samples. Percentage of viable cells was assessed after Annexin/7AAD-staining by flow cytometer. Data are presented as individual values with mean value as center of error bar ± standard deviation. For significance testing ordinary one-way ANOVA (S1.D-F) and two-way ANOVA (S1.B, C) with Dunnet posthoc test was used. NK cells were coincubated in media or media supplemented with 25% of different ascites samples for 24h. Viability of NK cells was quantified by Annexin/7AAD staining by using flow cytometry. ns (non-significant), * (p<0.05), ** (p<0.01), *** (p<0.001), **** (p<0.0001).


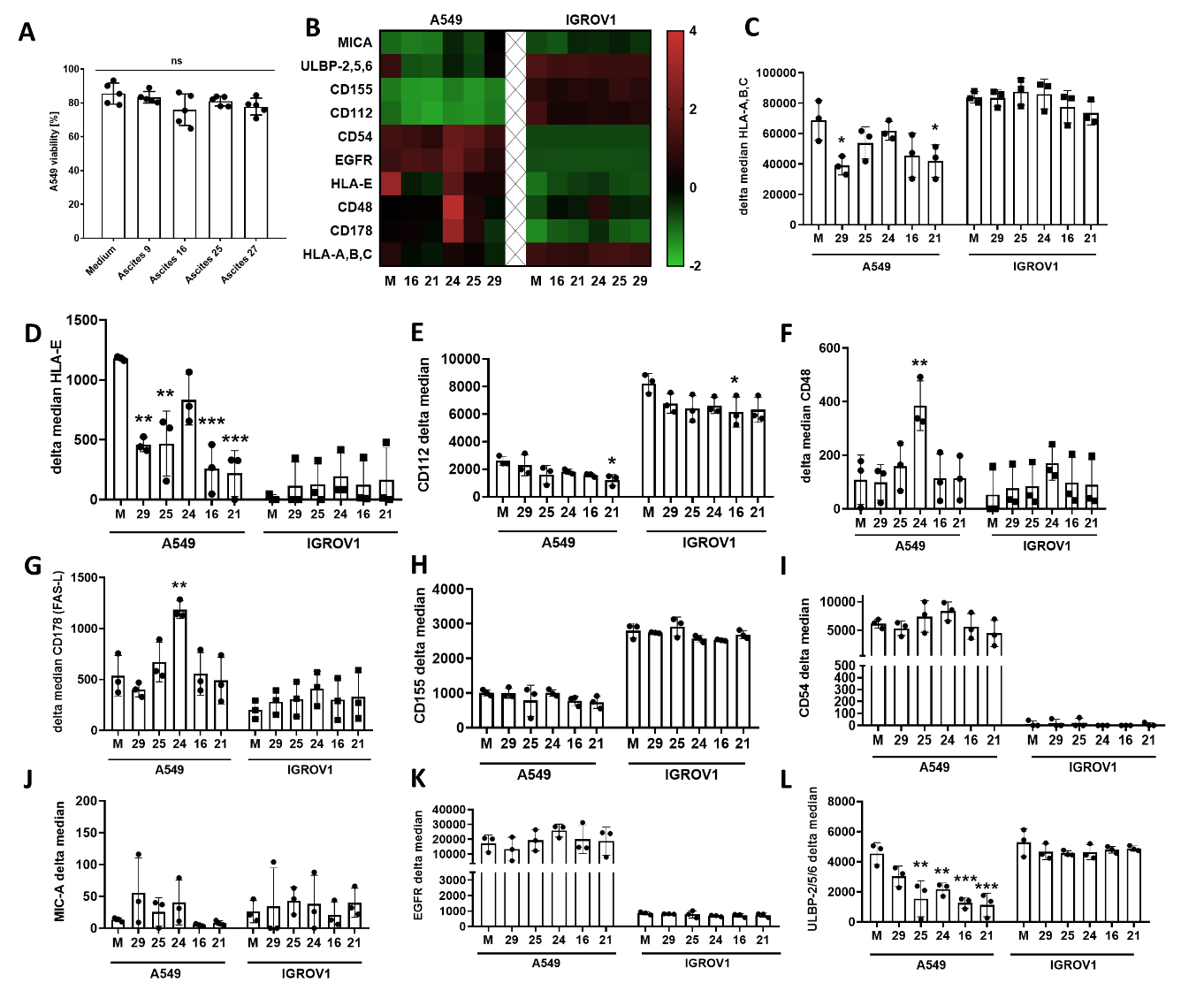


**Supplementary Figure 2. – Malignant ascites does not affect viability and surface marker expression of different EGFR-positive cancer cells.** Different types of EGFR-positive cancer cells were co-incubated with 25% ascites-supplemented media or media control for 24 hours. **(A) Percentage of viable A459 cells** after Annexin/7AAD-staining by flow cytometer**(B)**Heat map of receptor expression showing a summary of all surface marker expression changes in IGROV1 and A459 cell lines after treatment with different ascites samples after 24 hours.  Color intensity of individual fields corresponds to calculated z-score of marker expression. Surface marker expressions portrayed in heat map were shown as individual graphs: **(C)** HLA-A,B,C **(D)** HLA-E **(E)** CD112 **(F)** CD48 **(G)** CD178 **(H)** CD155 **(I)** CD54 **(J)** MICA **(K)** EGFR **(L)** ULBP-2,5,6. Data are presented as individual values with mean value as center of error bar ± standard deviation. For significance testing ordinary one-way ANOVA (S2.A, C-L) with Dunnet posthoc test was used. * (p<0.05), ** (p<0.01), *** (p<0.001).


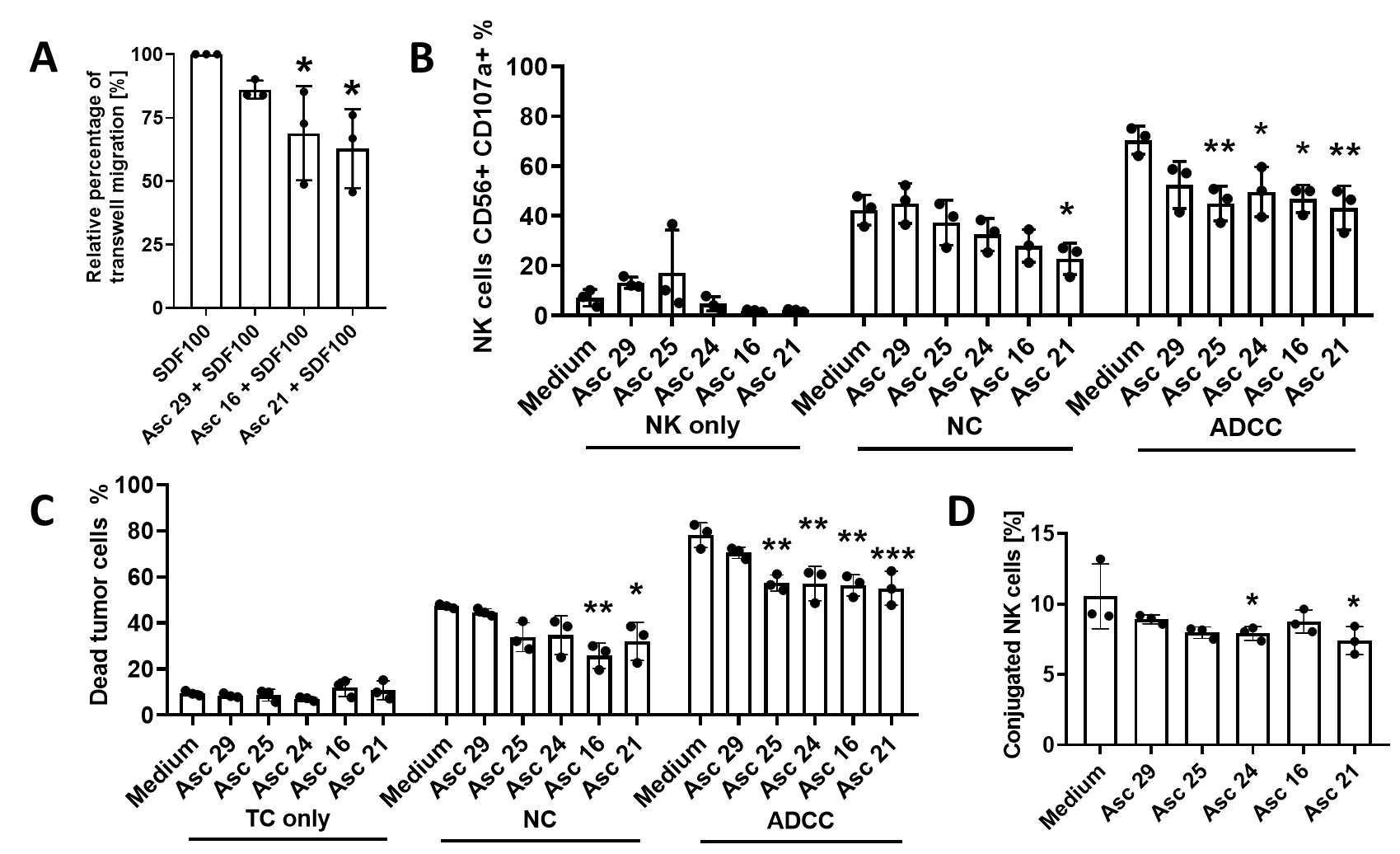


**Supplementary Figure 3. – Malignant ascites impairs different effector functions in IL2 stimulated NK cells. (A) Migration of NK cells in presence of ascites.** NK cells were added to the top of the transwell insert. Ascites samples were added to the bottom of the insert in 1:4 ratio. After 3 hours of SDF-1a, induced migration cells were collected and measured by flow cytometry. **(B and C)** **Cytotoxicity and tumor cell lysis of stimulated NK-cells directed against A549-cells in presence of ascites.** IL2 activated NK cells were coincubated in 1:1 ratio with A549 lung adenocarcinoma cells with or without Cetuximab (1 µg/ml). 25% ascites supplemented media or media control was added. **(B)** After 6 hours percentage of CD107a-positive NK cells were measured by flow cytometry. Left column shows NK control, middle column shows NC and right column ADCC. **(C)** After 24 hours, the tumor killing was assessed after 7AAD/Annexin-staining in the flow cytometer. Relative percentage of dead tumor cells in absence of Cetuximab (middle column, NC-condition) or presence of Cetuximab (right column, ADCC-condition) and tumor cells only for control (left column) is shown. **(D) Conjugation formation of stimulated NK cells in presence of ascites.** IL2-activated NK cells and IGROV1 cells were mixed in 4:1 effector to target ratio in presence of 1 µg/ml soluble Cetuximab in either normal cell culture media or supplemented with 25% of OC ascites. After 45 minutes of coincubation, percentage of the conjugated NK cells was measured by flow cytometry. Data are presented as individual values with mean value as center of error bar ± standard deviation. For significance testing ordinary one-way ANOVA (S3.A-D) with Dunnet posthoc test was used. ns (non-significant), * (p<0.05), ** (p<0.01), *** (p<0.001).


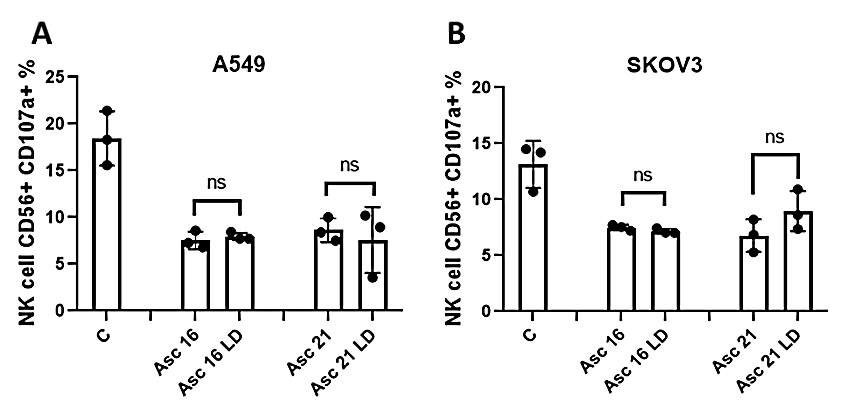


**Supplementary Figure 4. –** **Activated charcoal delipidation of ascites does not restore cytotoxic NK cell activity.** Ovarian cancer ascites was coincubated with activated charcoal for 1 hour and stirred magnetically, after which charcoal was removed by centrifugation. NK cells were coincubated in 1:1 ratio with EGFR-positive cancer cells in presence of various ascites samples and ADCC-inducing antibody Cetuximab (1 µg/ml). Percentage of CD107a-positive NK cells was determined after 6 hours by flow cytometry and was normalized to medium control. **(A) and (B)** NK cell mediated ADCC against **(A)** A549 or **(B)** SKOV3 in the presence of medium, untreated ascites and lipid-depleted ascites (LD). Paired t-test was used to assess the significance. ns – no significance.

**
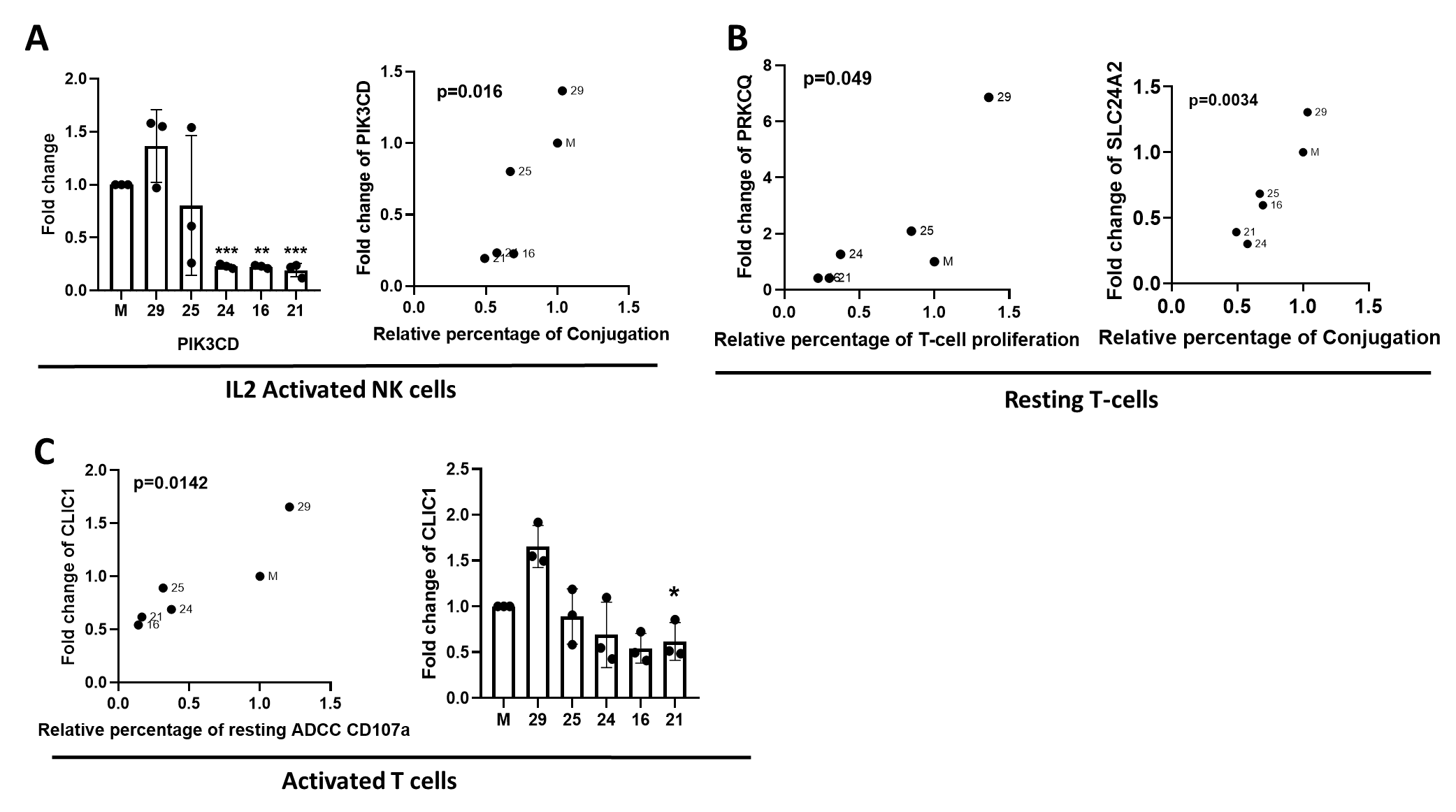
**

**Supplementary Figure 5. – Malignant ascites alters expression of signal transduction molecules and electrolyte channels in immune cells. (A)** Effects of ascites on IL2-activated NK cell gene expression. PI3K is significantly downregulated by suppressive malignant ascites (left). Significant Pearson correlation of PI3K to NK-tumor cell conjugation (right). **(B)** Effects of ascites on resting T cell gene expression. Significant Pearson correlation of PRK to T-cell proliferation inhibition (left), and SLC24A2 to NK-TC-conjugation (right). **(C)** Effects of ascites on activated T cell gene expression. Significant Pearson correlation of CLIC1 to ADCC of resting NK cells (left), significant impact of malignant ascites on CLIC1 (right). Data are presented as individual values with mean value as center of error bar ± standard deviation. For significance testing ordinary one-way ANOVA with Dunnet posthoc test (S5.A (left), C right)) and two-tailed Pearson correlation were used (S5.A (left), B, C (right). ** (p<0.01), *** (p<0.001).


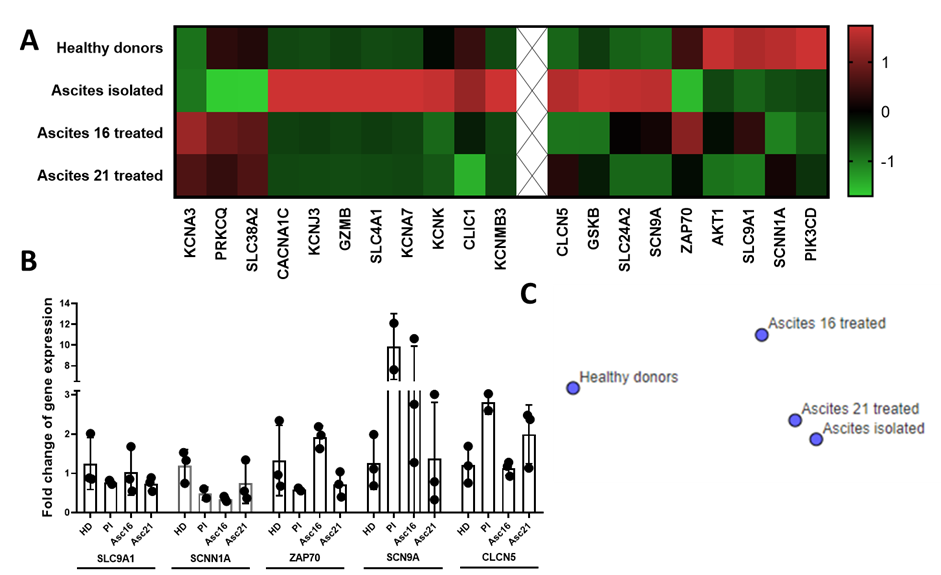


**Supplementary Figure 6. – Healthy donor T cells treated with ascites in vitro show similar transcriptional profile as patient-isolated T cells from ascites.** Resting T cells were exposed either to medium (HD) or 25% ascites supplemented medium (Asc) for 24 hours before proceeding with RNA isolation. RNA Isolation of patient ascites T cells (PI) was performed directly after cell isolation. For calculation of fold-change the gene expression was normalized to housekeeping gene and the mean expression of the indicated genes in the HD group. **(A)** Heatmap overview showing transcript expressions comparison between HD T cells in medium, two ascites-treated healthy donor T cell samples and T cells isolated from patient ascites. **(B)** Bar graph depicting gene transcript expression as determined by RNAseq. **(C)** t-SNE clustering of transcript expressions in healthy donor T cells, T cells from patient ascites and two ascites-treated healthy donor T cells.

| **Patient**  **No.** | **FIGO/ UICC** | **Neoplastic grading** | **Cancer type** | **Histology** | **Time of**  **diagnosis** | **Survival** |
| --- | --- | --- | --- | --- | --- | --- |
| **1.** | ---- | G3 | Ovarian | High-grade serous-papillary | Recurrence | 1Y 2M 10D |
| **2.** | ---- | G2 | Ovarian | High-grade serous | Recurrence | 6Y 2M 12D |
| **3.** | ---- | G2 | Ovarian | High-grade serous | Recurrence | 2Y 5M 20D |
| **4.** | IV | G3 | Ovarian | Signet ring cell adenocarcinoma | Initial | 13D |
| **5.** | ---- | G3 | Ovarian | High-grade serous-papillary | Recurrence | 8M 20 D |
| **6.** | IIIc | G2 | Ovarian | High-grade serous | Initial | Unknown |
| **7.** | ---- | G3 | Ovarian | High-grade serous | Recurrence | 11M 20 D |
| **8.** | IV | G3 | Ovarian | High-grade serous-papillary | Initial | 1Y 7M 2D |
| **9.** | IV | G3 | Ovarian | High-grade serous-papillary | Initial | 7Y 5M 2D |
| **10.** | III | G2 | Ovarian | Small cell hypercalcemic carcinoma | Initial | 1Y 0M 2D |
| **11.** | IV | G3 | Ovarian | Invasive mucinous | Initial | 3M 8D |
| **12.** | IV |  | B-cell Lymphoma | Diffuse large cell | Initial | Unknown |
| **13.** | IV | G3 | Ovarian | High-grade serous | Initial | Unknown |
| **16.** | IIIc | G3 | Ovarian | High-grade serous | Initial | 1M 15D |
| **18.** | IV | G2 | Colon carcinoma | Adenocarcinoma | Initial | 2Y 8M 4D |
| **19.** | IV | G3 | Ovarian | Invasive mucinous | Initial | 1Y 0M 6D |
| **20.** | IIIc | G3 | Ovarian | High-grade serous | Initial | 7M 21D |
| **21.** | IV | G3 | Unknown | Invasive adenocarcinoma | Initial | 1Y 10M 23D |
| **22.** | IIIc | G3 | Ovarian | High-grade serous-papillary | Initial | 1Y 5M |
| **24.** | IIIc | G2 | Ovarian | High-grade serous-papillary | Initial | 5D |
| **25.** | IIIc | G3 | Ovarian | High-grade serous | Initial | Unknown |
| **26.** | IV | G2 | Ovarian | High-grade serous-papillary | Initial | 7M 8D |
| **27.** | IIIc | G3 | Ovarian | High-grade serous-papillary | Initial | 6Y 0M 8D |
| **28.** | IIIc | G3 | Ovarian | High-grade serous-papillary | Initial | Unknown |
| **29.** | ---- | ----- | Congestive heart failure, benign diagnosis | | ---- | Still alive |
| **30.** | IIIc | G3 | Ovarian | High-grade serous-papillary | Initial | Unknown |
| **32.** | IIIc | G3 | Ovarian | High-grade serous | Initial | Still alive |
| **33.** | ---- | G3 | Ovarian | High-grade serous | Recurrence | Still alive |

**Supplementary Table 1. – Patient characteristics from ascites donors.** Table contains patient cohort information starting from left to right: Patient/Ascites number designation, FIGO/UICC cancer staging, histological grade, cancer tissue and type classification, timepoint and survival time since ascites collection.

| **Primer pair** | **Sequence ( 5´ - 3´ )** | | **Amplicon (bp)** |
| --- | --- | --- | --- |
|  |  | |  |
| **hu_CACNA1C_NM_000719** | forward | GCAGGAGTACAAGAACTGTGAGC | 143 |
|  | reverse | CGAAGTAGGTGGAGTTGACCAC |  |
| **hu_CLCN5_NM_000084** | forward | GTATCTGTAGCCTTTGGAGCACC | 105 |
|  | reverse | GGCAGCAAAGAATGAACGCCAC |  |
| **hu_CLIC1_NM_001288** | forward | CCTGCTGTATGGCACTGAAGTG | 120 |
|  | reverse | GCTGTGTTGGACTCAGGGTTCA |  |
| **hu_SCNN1A_NM_001038** | forward | GTGCCTACATCTTCTATCCGCG | 110 |
|  | reverse | GTCTGAGGAGAAGTCAACCTGG |  |
| **hu_SLC24A2_NM_020344** | forward | CCATCCAGTGATGCTTCAGAACC | 141 |
|  | reverse | CGTGACTTGCTTGCGGGTTTCA |  |
| **hu_SLC4A1_NM_000342** | forward | CTGCTGGTGTTTGAGGAAGCCT | 102 |
|  | reverse | CACCAGCAGGATGAGCCAGAAG |  |
| **hu_SCN9A_NM_002977** | forward | GTGGAAGGATTGTCAGTTCTGCG | 140 |
|  | reverse | GCCAACACTAAGGTGAGGTTACC |  |
| **hu_SLC9A1 _NM_003047** | forward | GAACTGGACCTTCGTCATCAGC | 109 |
|  | reverse | GGTCAGCTTCACGATACGGAAC |  |
| **hu_KCNA3_NM_002232** | forward | CGGTGTCTTGACCATCGCATTG | 131 |
|  | reverse | AAGAGGAGAGGTGCTGGCAACT |  |
| **hu_KCNA7_NM_031886** | forward | TCGAGACGCTGCCTGACTTCC | 152 |
|  | reverse | CACCACGAAGAACGGGTCATTG |  |
| **hu_KCNMB3_NM_014407** | forward | TCAGCCATCCAGGTCAGAAAGC | 98 |
|  | reverse | TCTATCTTGGTGGCACTTAGGTG |  |
| **hu_KCNJ3_NM_002239** | forward | GATCTCCATGAGGGACGGAAAAC | 131 |
|  | reverse | GAAGGAACTCACCCTCAGGTGT |  |
| **hu_KCNK3_NM_002246** | forward | GGCTCCTTCTACTTCGCCATCA | 137 |
|  | reverse | CTCTGGAACATGACGAGCGTGA |  |
| **hu_ORAI1_NM_032790** | forward | AGGTGATGAGCCTCAACGAGCA | 151 |
|  | reverse | AGTCGTGGTCAGCGTCCAGCT |  |
| **hu_ZAP70_NM_207519** | forward | CACTACGCCAAGATCAGCGACT | 139 |
|  | reverse | GGCTGGAGAACTTGCGGAAGTT |  |
| **hu_PRKCQ_NM_006257** | forward | GCATCCGTTTCTGACGCACATG | 133 |
|  | reverse | CGCTCTGGAAAGGTCGAACTTG |  |
| **hu_PIK3CD_NM_005026** | forward | TGCCAAACCACCTCCCATTCCT | 160 |
|  | reverse | CATCTCGTTGCCGTGGAAAAGC |  |
| **hu_GZMB_NM_004131** | forward | CGACAGTACCATTGAGTTGTGCG | 122 |
|  | reverse | TTCGTCCATAGGAGACAATGCCC |  |
| **hu_GSK3b_NM_002093** | forward | CCGACTAACACCACTGGAAGCT | 150 |
|  | reverse | AGGATGGTAGCCAGAGGTGGAT |  |
| **hu_AKT1_NM_005163** | forward | TGGACTACCTGCACTCGGAGAA | 154 |
|  | reverse | GTGCCGCAAAAGGTCTTCATGG |  |

**Supplementary Table 2. – List of all primers used for qPCR gene expression experiments.** Table contains from left to right: primer designation and NCBI Reference Sequence, primer direction and sequence, product (amplicon) length.

|  | **Relative percentage of effector function of NK cells** | | | |
| --- | --- | --- | --- | --- |
|  | **NC CD107a** | **ADCC CD107a** | **NC lysis** | **ADCC lysis** |
| **Asc No.** | **Extremely strong inhibitory ascites (CD107a<25%)** | | | |
| **21** | 4.9 | 9.4 | 44.6 | 71.2 |
| **16** | 3.7 | 11.0 | 25.4 | 51.7 |
|  | **Strong inhibitory ascites (50%>CD107a>25%)** | | | |
| **22** | 12.6 | 28.7 | 70.1 | 94.6 |
| **25** | 7.0 | 30.7 | 57.5 | 75.81992 |
| **19** | 20.8 | 32.7 | 52.0 | 71.0 |
| **20** | 12.7 | 35.3 | 66.9 | 97.3 |
| **30** | 11.7 | 39.6 | 75.2 | 101.5 |
| **11** | 21.7 | 43.9 | 63.8 | 93.8 |
| **24** | 8.0 | 44.3 | 40.3 | 88.5 |
| **26** | 8.7 | 44.9 | 59.0 | 89.2 |
|  | **Medium inhibitory ascites (75%>CD107a>50%)** | | | |
| **27** | 14.7 | 51.7 | 90.6 | 105.2 |
| **32** | 19.0 | 55.6 | 65.6 | 93.8 |
| **18** | 21.0 | 56.6 | 64.0 | 96.9 |
| **13** | 14.2 | 61.6 | 73.9 | 102.6 |
| **8** | 20.0 | 61.8 | 79.2 | 104.0 |
| **2** | 7.0 | 68.3 | 52.4 | 88.0 |
| **9** | 9.0 | 68.3 | 52.7 | 83.1 |
| **7** | 18.4 | 69.5 | 79.2 | 104.0 |
| **5** | 17.5 | 70.3 | 76.9 | 102.3 |
|  | **Weak inhibitory ascites (100%>CD107a>75%)** | | | |
| **3** | 22.5 | 79.3 | 104.3 | 104.3 |
| **1** | 46.3 | 80.3 | 91.3 | 104.9 |
| **10** | 22.0 | 81.2 | 72.1 | 96.8 |
| **4** | 102.0 | 83.4 | 77.3 | 89.4 |
|  | **Stimulatory ascites (CD107a>100%)** | | | |
| **29** | 71.3 | 116.0 | 81.4 | 102.4 |

**Supplementary Table 3. – List of ascites samples ordered according to their inhibitory power.** Table contains overview of used patient ascites samples ranked into five different categories depending on its effects on average ADCC NK cell degranulation in coculture system. Listed from left to right: relative percentage of NK cell natural toxicity degranulation and **cetuximab induced ADCC degranulation**, relative percentage of IGROV1 lysis in natural toxicity and ADCC conditions. The average relative percentage of degranulation (CD107a) or lysis was calculated after four independent experiments by normalizing ascites condition samples to appropriate medium controls. Each experiment was performed using NK cells isolated from different healthy donors.
